# Comprehensive Quantitative Profiling of Less Polar Lipids in Human Plasma Using Validated Reversed-Phase UHPSFC/MS/MS

**DOI:** 10.1021/acs.analchem.5c04668

**Published:** 2025-11-24

**Authors:** Zuzana Lásko, Veronika Šubrtová, Ondřej Peterka, Robert Jirásko, Michal Holčapek

**Affiliations:** Department of Analytical Chemistry, Faculty of Chemical Technology, University of Pardubice, Studentská 573, 53210 Pardubice, Czech Republic

## Abstract

The quantitative analysis of lipids is challenging due to their structural diversity and the coexistence of numerous isomers in biological samples. Here, we present a robust and validated method based on reversed-phase ultrahigh-performance supercritical fluid chromatography-tandem mass spectrometry (RP-UHPSFC/MS/MS) for the high-throughput profiling of less polar lipids in human plasma. The sample preparation workflow involves an initial Folch-based extraction, followed by chemical derivatization with benzoyl chloride, and the final liquid–liquid extraction into hexane to isolate less polar acylated analytes. This strategy enables the sensitive profiling of six major lipid classes (TG, DG, MG, SE, ST, and FA) in positive ion mode in less than 18 min. The use of two C18 columns in series provides high chromatographic resolution, sufficient to separate isomeric species, including *cis*/*trans* and positional isomers of double bonds in fatty acids. The method was rigorously validated for five lipid classes, except free FA, and the implementation of response factors was shown to be essential for the accurate quantification of sterol esters. A total of 147 lipid species were quantified in NIST SRM 1950 human plasma, demonstrating its suitability for large-scale lipidomic studies focused on less polar lipids.

## Graphical abstract

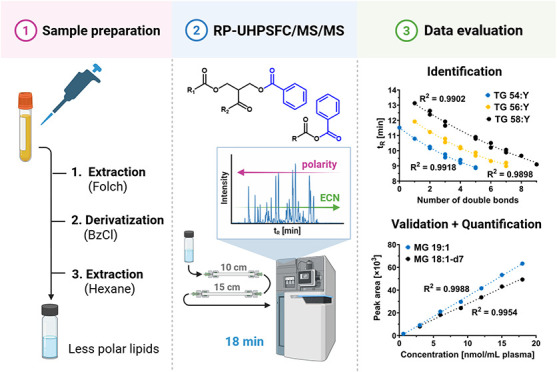

## Introduction

Lipidomics is a field dealing with the determination of lipids in biological systems at a defined time. Within this complex landscape, a group of lipids centered around a glycerol or sterol backbone plays fundamental roles in energy storage, membrane structure, and signaling pathways.^1^ For the purpose of this study, this group, which includes triacylglycerols (TG), diacylglycerols (DG), monoacylglycerols (MG), free sterols (ST), sterol esters (SE), and their metabolically related free fatty acids (FA), will be referred to as “less polar lipids”. While the collective roles of these classes in maintaining cellular homeostasis are established, the distinct functions of individual lipid species remain largely unresolved. This knowledge gap stems from profound analytical challenges, including the vast structural diversity and the coexistence of numerous isomers within complex biological matrices. Addressing this challenge requires highly efficient analytical methods with the resolving power to accurately distinguish and quantify these lipids in their endogenous environment.^2,3^

Due to its high sensitivity, specificity, and ability to detect a wide range of large lipid species across various classes, mass spectrometry (MS) has become an indispensable tool for the comprehensive analysis of large lipid data sets. In lipidomics, two principal strategies are commonly employed: direct infusion MS, and chromatographic separation followed by MS detection.^4^ While the direct infusion offers rapid data acquisition, it often suffers from ion suppression and limited ability to resolve isomeric species.^5,6^ In contrast, the chromatographic separation prior to MS detection significantly improves both qualitative and quantitative performance, particularly when analyzing complex biological matrices, such as plasma or serum.^7^

Among chromatographic techniques, reversed-phase (RP) ultrahigh-performance liquid chromatography (UHPLC)^8–10^ and hydrophilic interaction liquid chromatography (HILIC)^11–14^ are widely used in lipidomics. RP-UHPLC is effective for separating less polar and moderately polar lipids. HILIC offers good separation of polar lipid classes, but both techniques struggle with subtle isomeric differences and may require complementary methods.^2^ Ultrahigh-performance supercritical fluid chromatography (UHPSFC) has recently gained interest in lipidomics due to short run times, separation efficiency, and high reproducibility, making it suitable for high-throughput analysis.^15–19^ The use of a supercritical mobile phase, typically CO_2_, provides low viscosity and high diffusivity, resulting in faster separations compared to UHPLC.^20^ UHPSFC separations may resemble RP or HILIC modes, similar to UHPLC. However, the retention mechanism is not entirely identical. Therefore, a more accurate description of the retention behavior in UHPSFC would be ″RP-like″ or ″HILIC-like″. Nevertheless, for the sake of simplicity, we use the term ″RP-UHPSFC″ throughout this manuscript to refer to the RP-like retention mechanism.

RP-UHPSFC coupled to electrospray ionization MS has emerged as a powerful alternative particularly, for nonpolar lipid species, and extends the advantages of conventional SFC, offering high separation efficiency and improved resolution of isomeric lipid species.^21^ This technique allows for the simultaneous separation of lipid species based on their polarity and structural characteristics, such as fatty acyl chain length, degree of unsaturation, and the positional and geometrical isomerism of double bonds.^22–25^ Furthermore, its compatibility with electrospray ionization MS allows for sensitive and selective detection across a wide range of less polar lipid species. This makes RP-UHPSFC/MS well suited for the comprehensive characterization of TG, SE, and structurally related less polar lipid species, which are often difficult to separate and quantify using traditional RP-UHPLC. While not yet as widely adopted or validated for routine quantitation as RP-UHPLC, RP-UHPSFC/MS shows a strong potential for detailed lipid species analysis, particularly in cases where the structural resolution is critical.^26^

The primary objective of this study is to develop a novel and highly reproducible RP-UHPSFC method for the analysis of less polar lipids in plasma samples. The method is used in combination with the chemical derivatization using benzoyl chloride (BzCl),^27^ which enhances the detection sensitivity and improves analytical performance for lipid species with low abundance and/or low ionization efficiency. The chromatographic system employs two C18 columns connected in series to achieve high-resolution separation and is coupled to high-resolution MS, which enables accurate mass determination and tandem mass spectrometry (MS/MS) experiments for reliable lipid species identification. This integrated approach provides robust and detailed profiling of less polar lipid species, offering an advanced tool for clinical and biomedical investigations.

## Experimental Section

### Chemicals and Standards

2-propanol (IPA), acetonitrile (ACN), methanol (MeOH), water, hexane (Hex), butanol, ammonium acetate (AmAc), acetic acid (all LC/MS grade), and ammonium carbonate (≥30.0% NH_3_ basis) were purchased from Honeywell (Riedel-de Han, Hamburg, Germany). Benzoyl chloride (BzCl, 99%) and pyridine (Pyr; ≥99.9%) were purchased from Sigma-Aldrich (St. Louis, MO) and LiChrosolv chloroform (HPLC grade) stabilized with 2-methyl-2-butene was purchased from Merck (Darmstadt, Germany). Carbon dioxide of 4.5 grade (99.995%) was obtained from Messer Group (Bad Soden, Germany). Deionized water was prepared by a Milli-Q Reference Water Purification System (Molsheim, France). Reference lipid standards, including both species endogenous to plasma and nonendogenous species (i.e., shorter chain fatty acyls (C12 or C13), deuterated lipids) were purchased from Avanti Polar Lipids (Alabaster, AL) and Nu-Check Prep (Elysian, MN). Deuterated and other nonendogenous lipids used as internal standards (IS), including FA 19:1, FA 13:0, FA 18:1-d9, Chol-d7, CE 12:0, CE 16:0-d7, MG 19:1, MG 18:1-d7, DG 18:1/18:1 d5, DG 15:/18:1-d7, TG 12:0/12:0/12:0, and TG 15:0/18:1-d7/15:0, were prepared as a dedicated IS mixture (ISmix). The compositions and final concentrations of the reference standard mixture and ISmix are shown in Tables S1 and S2, respectively.

### Plasma Samples

Pooled plasma samples, used for optimization of the extraction protocol, UHPSFC/MS/MS method development, validation, and lipid identification, were prepared by combining aliquots from 200 human plasma samples collected from healthy volunteers (age 44–66 years and body mass index of 18–39). Samples of 100 male and 100 female volunteers were obtained from the Transfusion Department, University Hospital Olomouc, Czech Republic. The study was approved by the Ethics Committee of the University Hospital Olomouc and the Faculty of Medicine, Palacký University Olomouc (reference number: 117/23), and all subjects signed an informed consent. All plasma samples were stored at −80 °C. NIST Standard Reference Material 1950 human plasma was used for the quantitation of less polar lipids.

### Sample Preparation

The modified Folch extraction^26^ protocol was used for the lipid extraction. Pooled plasma (25 μL) was spiked with 20 μL of ISmix and combined with 2 mL of chloroform and 1 mL of MeOH. The mixture was vortexed for 1 min and subsequently ultrasonicated at ambient temperature for 15 min. Afterward, 600 μL of 250 mM ammonium carbonate buffer was added, and the mixture was stirred for 5 min at 560 rpm (KS 130 shaker, IKA, Staufen, Germany), followed by ultrasonication for 15 min. Then, the solution was centrifuged at 3462*g* (Hettich EBA 20) for 3 min, and the collected organic phase was evaporated under a gentle stream of nitrogen.

The reaction using BzCl was optimized and applied for the derivatization of the residue.^27^ The dried lipid extract was redissolved in 335 μL of Pyr:ACN (1:9, v/v) and stirred for 5 min at 320 rpm. Subsequently, 120 μL of BzCl:ACN (1:9, v/v) was added and stirred at 320 rpm for an additional 10 min. The reaction was stopped by adding 600 μL of 250 mM (NH_4_)_2_CO_3_. To extract the derivatized nonpolar lipids, 2 mL of Hex was added to the mixture, which was then stirred for 5 min at 640 rpm and centrifuged (3462*g*) for 3 min. The Hex layer was collected and evaporated under a gentle stream of nitrogen. The residue was stored at −80 °C or dissolved in 250 μL CHCl_3_:MeOH (1:1, v/v) for the UHPSFC/MS analysis. The solution was 40-times diluted with CHCl_3_:MeOH (1:1, v/v) prior to injection. All steps of sample preparation were performed under ambient temperature. The whole process of extraction is shown in Figure 1.

**Figure 1:**
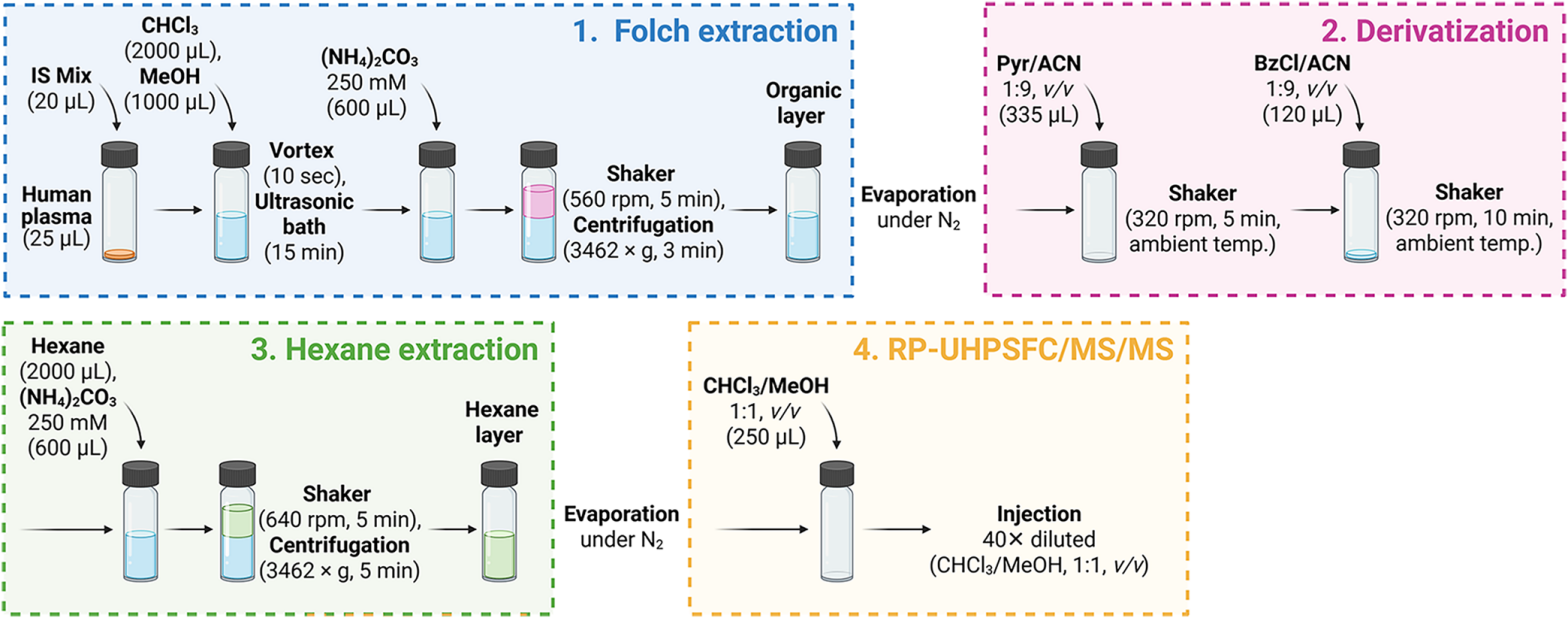
Schematic diagram of extraction and derivatization protocols of less polar lipids from human plasma sample measured by the RP-UHPSFC/MS/MS method. Figure was created using BioRender.

### RP-UHPSFC/MS Conditions

The experiments were performed on a supercritical fluid chromatograph Acquity UPC^2^ (Waters, Milford, MA) with the following conditions: two Viridis HSS C18 SB columns (100 × 3.0 mm; 1.8 μm and 150 × 3.0 mm; 1.8 μm, Waters) connected in series, column temperature 40 °C, backpressure 1800 psi (124.2 bar), flow rate 1 mL/min, injection volume 1 μL, and mobile phase gradient: 0 min – 2% B, 17 min – 13% B, 17.1 min – 2% B, 18 min – 2% B, where the phase A was supercritical CO_2_ (scCO_2_), and the phase B was MeOH with 0.1% acetic acid. To enhance ionization, the makeup solvent consisted of MeOH with 0.1% acetic acid with a flow rate of 0.1 mL/min was used. The total run time of the method was 18 min, including 1 min column equilibration.

The quadrupole time-of-flight mass spectrometer Synapt G2-Si QTOF (Waters) was used with the following parameters: capillary voltage 3 kV, sampling cone 20 V, source offset 90 V, ion source temperature 150 °C, desolvation temperature 500 °C, cone gas flow 50 L/h, desolvation gas flow 1000 L/h, acquisition range *m*/*z* 150–1000 in the positive ion mode, scan time 2 Hz, and continuum profile mode. MS/MS experiments were performed with a collision energy of 30 eV and scan time of 10 Hz. Leucine enkephalin was used as the lock mass for an accurate mass calibration.

### Data Processing

MassLynx software (Waters) was used for the method acquisition and data evaluation, with subsequent noise reduction applied via the Waters Compression Tool. To achieve higher mass accuracy, a lock mass correction was applied, and data were converted from continuum to centroid mode using the Accurate Mass Measure tool in MassLynx. Peak areas were extracted using the TargetLynx tool, with tolerances set at ± 15 mDa for *m*/*z* and ± 0.2 min for retention times. MS/MS spectra were acquired without the application of a lock mass correction. The plots were constructed using GraphPad Prism (v 10.2.1, GraphPad Software, Boston, MA). Quantitative results were obtained using the Skyline software^28^ (v. 23.1.0.268, MacCoss Lab., University of Washington, Seattle, WA). Graphical abstract and Figure 1 were created using BioRender (https://biorender.com).

## Results and Discussion

### Extraction of Less Polar Lipids

The main objective of this study was to develop a selective and efficient workflow for the extraction and analysis of less polar lipid classes from human plasma. Although these lipid species can be analyzed by using commonly applied chromatographic techniques, such approaches often introduce significant limitations when applied to this lipid group. Basic HILIC^11,29^ and UHPSFC^16,17,30^ methods do not provide sufficient structural resolution at the lipid species level. In the case of RP-UHPLC,^31,32^ which is commonly used for comprehensive lipid profiling, the simultaneous analysis of polar and nonpolar lipids results in compromised resolution of individual isomers. Furthermore, the use of high concentrations of IPA in the mobile phase often leads to the fast and less repeatable elution of strongly retained nonpolar lipids from the chromatographic system, which subsequently affects the reproducibility and accuracy of their quantification. A possible solution involves the use of nonaqueous RP-UHPLC, which offers improved separation of nonpolar lipid species, especially TG. However, these approaches are often time-consuming and less suitable for high-throughput analyses. To overcome these limitations, we developed an alternative strategy based on RP-UHPSFC, combined with selective extraction and derivatization, to enhance the analytical performance for these lipid classes.

Conventional lipidomics extraction protocols, such as the Folch,^33^ Bligh–Dyer,^34^ or MTBE^35^ methods, are based on the extraction of a broad spectrum of lipids and generally lack selectivity for nonpolar lipids. These approaches often coextract large amounts of polar lipids, which can negatively impact ionization efficiency. For our targeted analysis of less polar species, including TG, DG, MG, FA, SE, and ST, we implemented a more selective sample preparation method, in which derivatization with BzCl^27^ was applied immediately after lipid extraction. BzCl reacts with free hydroxyl groups present in MG, DG, and ST, and the carboxylic group in FA, thereby increasing their hydrophobicity and influencing their chromatographic behavior. In contrast, TG and SE lack free functional groups and, therefore, remain unmodified. Our previous study^27^ suggests that other lipid subclasses containing free hydroxyl or amino groups are also amenable to benzoylation, however, they were not the focus of this work and were selectively removed from the samples by extracting the less polar lipids into Hex.

Initially, we evaluated a one-phase protein precipitation protocol using a butanol:methanol (1:1, v/v) mixture. However, this approach led to the formation of DG artifacts, likely resulting from the enzymatic hydrolysis of TG species. These artifacts interfered with data processing and interpretation; therefore, this protocol was excluded from further method development. A two-phase extraction using the modified Folch protocol^26^ was selected as the most suitable initial step for isolating lipids from plasma. All optimization steps for the sample preparation were performed by using pooled plasma samples spiked with 20 μL of the ISmix. To optimize the derivatization process, various reaction times (5, 10, 15, 30, and 60 min) were evaluated (Figure S1A). The original reaction time of 60 min could be reduced to 10 min without a loss of performance for the investigated lipid classes, as this duration provided results comparable to those obtained with longer reagent exposure. Conversely, the reaction time of 5 min was found to be insufficient.

The addition of ammonium carbonate during the extraction facilitates the hydrolysis of excess derivatization reagents, thereby minimizing the risk of contaminating the mass spectrometer with reagent residues. To evaluate the most effective termination strategy for stopping the derivatization reaction, three different extraction procedures were tested: (i) modified Folch extraction using CHCl_3_:MeOH:(NH_4_)_2_CO_3_ (2:1:0.6; v/v/v), (ii) Hex-MeOH-based extraction with Hex:MeOH:(NH_4_)_2_CO_3_ (2:1:0.6; *v/v/v*), and (iii) Hex-based extraction using Hex:(NH_4_)_2_CO_3_ (2:0.6; *v/v*). A comparison of these three extraction strategies is presented in Figure S1B. The results demonstrate that the protocol utilizing Hex:(NH_4_)_2_CO_3_ provided higher extraction efficiency compared to Hex:MeOH:(NH_4_)_2_CO_3_ and, importantly, was the only method that enabled effective recovery of the arising free FA mixed anhydrides. Therefore, this protocol was selected as being the most suitable for the selective extraction of less polar lipids. The modified Folch protocol was not selected due to insufficient selectivity for less polar lipids and coextraction of interfering polar compounds. The overall extraction workflow for less polar lipids from human plasma samples is illustrated in Figure 1.

### RP-UHPSFC/MS/MS Method Development

As previously mentioned, RP-UHPSFC was selected as the separation technique for the analysis of less polar lipid classes due to its advantages over conventional RP-UHPLC and class separation UHPSFC methods. UHPSFC operates with lower backpressure, which allows the use of higher flow rates and thus faster analysis while maintaining a high separation efficiency. In the initial optimization phase, one column (150 × 3.0 mm; 1.8 μm, Viridis HSS C18 SB) was employed with the mobile phase gradient: 0 min – 2% B, 12 min – 20% B, 12.5 min – 2% B, 13 min – 2% B. For optimization of chromatographic and MS parameters, 73 lipid standards (Table S1) after the derivatization procedure and/or derivatized pooled plasma samples spiked with ISmix (Table S2) were used.

The mobile phase composition is a key parameter influencing the separation of analytes in chromatography. In SFC, the mobile phase typically consists of scCO_2_ as the main component (phase A). An organic cosolvent, known as the modifier (phase B), is commonly incorporated into the mobile phase to increase its polarity and solvating power, enabling the effective elution of a wider range of more polar analytes. ScCO_2_ has physicochemical properties similar to Hex, which makes it well-suited for nonpolar analytes. The modifier typically consists of MeOH or ACN, often with a small amount of water and organic additives, which are commonly added to increase the elution strength, improve separation efficiency, and enhance ionization efficiency in the MS detection.^30^ Based on the literature data and previous laboratory experience, several modifiers were evaluated, including MeOH, MeOH/ACN (1:1; *v/v*), MeOH/ACN (1:9; *v/v*), and IPA. MeOH alone provided the best compromise between the chromatographic resolution and ionization efficiency. The use of the MeOH/ACN (1:9; *v/v*) mixture or pure IPA did not yield significant improvements and negatively affected peak shapes. The influence of solvents used as the modifiers on the separation of individual less polar lipid classes represented by lipid standards is shown in Figure S2.

The addition of water (0, 1, 2, 3, and 5%) to the modifier was also investigated to improve the peak shapes and resolution. Higher percentages of water led to reduced chromatographic resolution, peak broadening, and tailing (data not shown) for nonpolar lipids. Therefore, pure MeOH without added water was selected as the optimal modifier, in terms of retention times and peak shapes of analytes. Seven different concentrations of AmAc in MeOH (0, 0.5, 5, 10, 20, 30, and 50 mM) were tested to assess their impact on separation and ionization efficiency. Figure S3A shows that the highest peak areas and separation efficiency for selected lipid standards were obtained with pure MeOH. The only exception was Chol-d7, which showed slightly improved ionization at 0.5 mM AmAc; however, the difference compared to pure MeOH was small. Subsequently, the addition of 0.1% acetic acid (a commonly used concentration in lipidomics) was investigated and found to enhance the ionization efficiency for nearly all tested lipid standards, with the exception of Chol-d7 (Figure S3B). The variations in AmAc and acetic acid concentrations had no distinguishable effect on retention times or peak shapes. Based on all these findings, MeOH with 0.1% acetic acid was selected as the optimal modifier for further analyses.

The column temperature directly affects the density of scCO_2_ and, consequently, the elution power of the mobile phase. The retention behavior was evaluated at 40, 50, and 60 °C using derivatized plasma spiked with ISmix. The most effective separation was achieved at 40 °C, particularly for isobaric species. Higher temperatures led to a reduction in chromatographic resolution and broader peaks (Figure S4). Backpressure was evaluated at 1800, 2000, 2100, and 2500 psi (data not shown). Although higher back pressures accelerated elution, they also decreased the resolution of isomeric lipids. The pressure of 1800 psi provided the best balance between time of the analysis and resolution. Back pressures below 1800 psi caused system overpressure.

To further improve the resolution of isobaric compounds, the second column (100 × 3.0 mm; 1.8 μm, Viridis HSS C18 SB) was connected in series using the short metal capillary with the final mobile phase gradient described in the Experimental section. The advantage of employing two columns compared to a single column is demonstrated by the separation of TG 54:3 and FA 18:1 isomers in Figure S5. In UHPSFC/MS analysis, a splitter is routinely required because the high flow rates used in UHPSFC are not compatible with MS detection. To ensure the optimal electrospray ionization, a commercially available SFC-MS Splitter Kit (Waters, Milford, MA) was used, delivering a flow of makeup solvent with the same composition as the modifier (MeOH + 0.1% acetic acid). Since the addition of makeup solvent dilutes the analytes and may reduce sensitivity, the flow rate was minimized from 0.3 and 0.25 mL/min to 0.1 mL/min (data not shown) to maintain stable spray conditions without compromising detection. The ion source parameters were further optimized, with the source temperature of 150 °C and the desolvation temperature of 500 °C providing the best performance in terms of sensitivity and reproducible ionization across all lipid classes (Figure S6A,B).

### RP-UHPSFC/MS/MS lipid separation and identification

The separation of lipids in RP-UHPSFC mode is primarily determined by their polarity and, within individual lipid classes, by their equivalent carbon number (ECN).^31,32^ ECN is defined as the difference between the total number of carbon atoms (CN) in the fatty acyl chains and twice the number of double bonds (DB), following the equation ECN = CN – 2DB. The higher CN in fatty acyl chains increases retention times due to stronger hydrophobic interactions, whereas the presence of additional DB reduces retention. The derivatization using BzCl reduces the polarity of the analytes by transforming free hydroxyl-containing molecules into less polar derivatives. As a result, MG and DG exhibit chromatographic behavior similar to that of TG with shorter chain fatty acyl groups eluting in the order of MG, DG, and TG. Moreover, derivatives provided significantly higher responses compared to natural forms, increasing the sensitivity of their determination.^27^ Derivatized FA form anhydrides, which are efficiently ionized in positive ion mode, in contrast to their underivatized forms that typically require analysis in negative ion mode. These compounds, similar to SE and ST, elute later in the analysis due to their low polarity. TG elute throughout the entire chromatographic window due to the large range of ECN values. The separation of individual lipid classes in the derivatized human plasma sample is shown in Figure 2A, while Figure 2B demonstrates the retention behavior of 20 TG standards, differing in CN and DB number.

**Figure 2:**
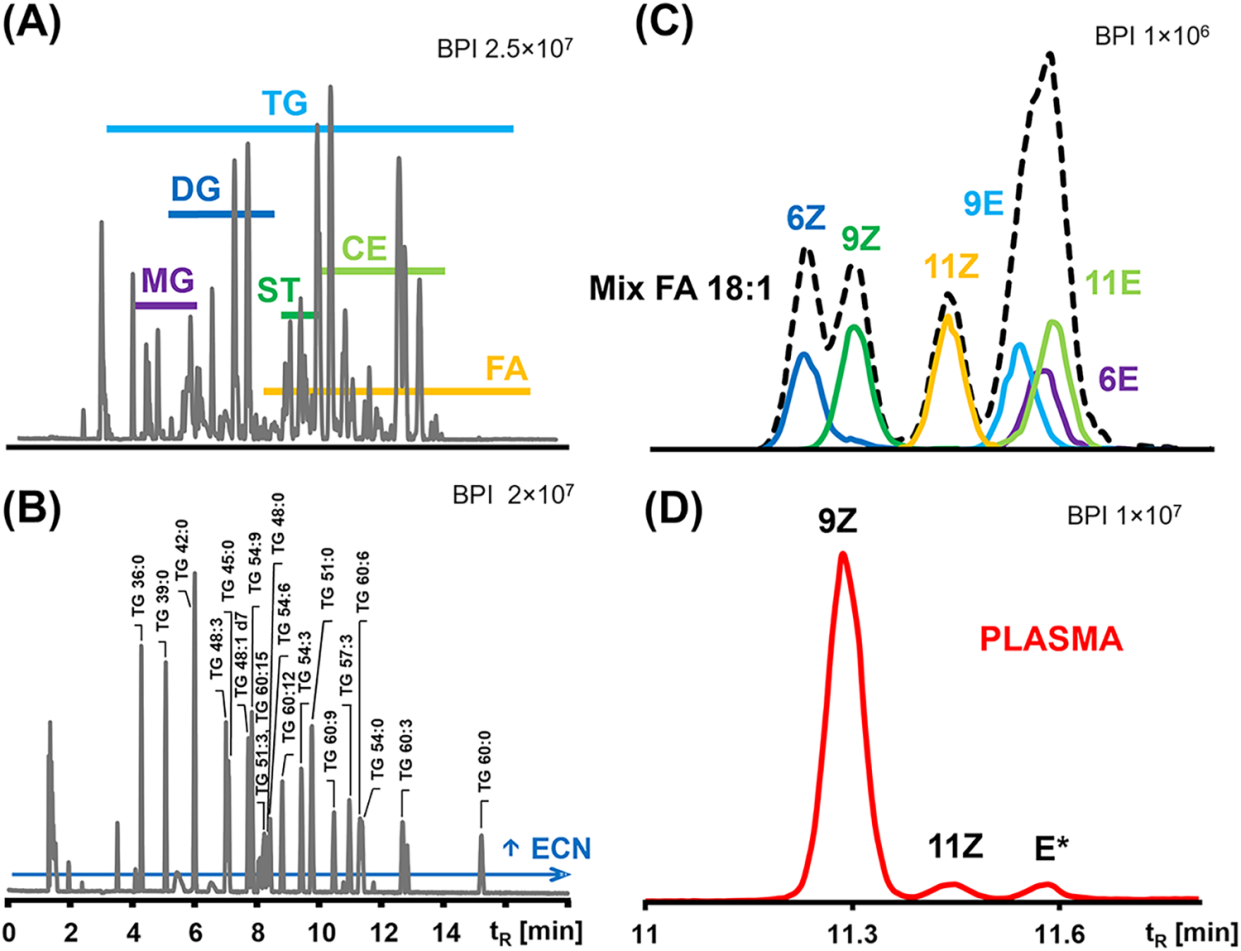
Base peak chromatograms of: (A) human plasma sample showing the retention of individual lipid subclasses, (B) triacylglycerol standard mixture showing the retention behavior in RP-UHPSFC chromatographic mode, (C) separation of FA 18:1 positional isomers in the standard mixture, and (D) separation of FA 18:1 positional isomers in the human plasma sample. E* denotes a tentatively identified *trans*-isomer.

The optimized RP-UHPSFC/MS/MS method enables the separation of isomeric compounds and positional isomers (Figure 2C). The six positional isomers of FA 18:1 (6Z, 9Z, 11Z, 6E, 9E, and 11E) standards were successfully separated. *Cis*-isomers eluted earlier due to their more compact, bent conformations that limit hydrophobic interactions, while *trans*-isomers, exhibiting more linear structures, were more retained and eluted closely to saturated analogs. A comparable retention behavior was observed for FA 18:3 standards, where 6Z,9Z,12Z eluted earlier than 9Z,12Z,15Z. These retention trends were applied to pooled plasma samples, where FA 18:1 (9Z), FA 18:1 (11Z), FA 18:1 (E*) (shown in Figure 2D), and FA 18:3 (6Z,9Z,12Z) with FA 18:3 (9Z,12Z,15Z) were confidently identified.

In the MS spectra, the most commonly observed ions for acylglycerols were [M + NH_4_]^+^ and [M + Na]^+^ adduct ions. For FA, the most abundant detected ion was the fragment ion [M + NH_4_ – C_7_H_6_O_2_]^+^, while ST provided the fragment ion [M + H – C_7_H_6_O_2_]^+^, which indicates the cleavage of benzoic acid from the derivatized molecule caused by in-source fragmentation. SE formed [M + Na]^+^ and [M + NH_4_]^+^ adducts, and the product ion at *m*/*z* 369 (already in the full-scan spectra) corresponds to the characteristic fragment of these compounds, specifically representing the cholesterol or lathosterol part. Although these analytes are most likely cholesteryl esters, which are known to be the most abundant SE in human plasma/serum, their exact identity cannot be unambiguously confirmed based on the current MS data. The fragmentation of MG and DG predominantly produced fatty acyl residue ions [RC = O + 74 + C_7_H_5_O]^+^ with the loss of H_2_O. TG fragmentation yielded [M + H R_i_COOH]^+^ ions, enabling the identification of the fatty acyl composition. Characteristic precursor and fragment ions of individual lipid classes in MS and MS/MS spectra are summarized in Table S3. Figure S7 illustrates the example of the MS/MS spectrum annotation of selected TG 54:5.

The retention behavior of lipids can be effectively demonstrated by constructing a graphical dependence between retention times and CN or DB number using the second-degree polynomial regression. Selected regressions are shown in Figure 3, with additional examples in Figure S8 (*t*_R_ vs CN) and Figure S9 (*t*_R_ vs DB number). In many cases, the correlation coefficient (*R* ^2^) exceeded 0.999, providing strong supporting information for lipid identification. Individual less polar lipid species were identified based on accurate masses (mass accuracy <5 ppm), characteristic MS/MS fragment ions, and homological dependences in the retention behavior of lipid species. In total, 156 lipid species from 6 less polar classes (TG, DG, MG, FA, SE, and ST) were identified in the pooled human plasma sample. Detailed information, including retention times, diagnostic ions, and mass accuracy, is summarized in Table S4. However, it is important to note that the reported number of TG species corresponds to the number of chromatographically resolved peaks and not to unique molecular entities. Due to coelution of multiple isomeric and isobaric TGs that share identical precursor masses but differ in fatty acyl chain combinations, each peak may represent several possible TG structures. Therefore, the actual number of TG species is likely underestimated and remains to be fully elucidated through advanced structural analysis. Lipid nomenclature and shorthand notation follow the updated guidelines by Liebisch et al.^36^ The number of identified lipid species was compared with previously published data, as summarized in Table 1. This comparison focused primarily on methods employing RP separation, but for a broader context, it also included results from comprehensive interlaboratory studies that utilized a variety of analytical approaches.^37,38^ Regarding SE and ST, our method identified a number of species comparable to most other approaches, with 15 SE and one ST, which aligns with most method reports. Although Quehenberger et al.^38^ reported a higher total number of ST, their data set represents a compilation from multiple targeted analytical strategies, including gas chromatography–mass spectrometry or other specialized methodology optimized specifically for sterol analysis. Therefore, their results are not directly comparable with those of our current method. The number of TG species identified in our study is also consistent with previously reported RP methods. Ovčačíková et al.^32^ reported a slightly higher number of TG species, however, this was achieved at the expense of significantly longer analysis time (160 min vs 18 min).

**Figure 3:**
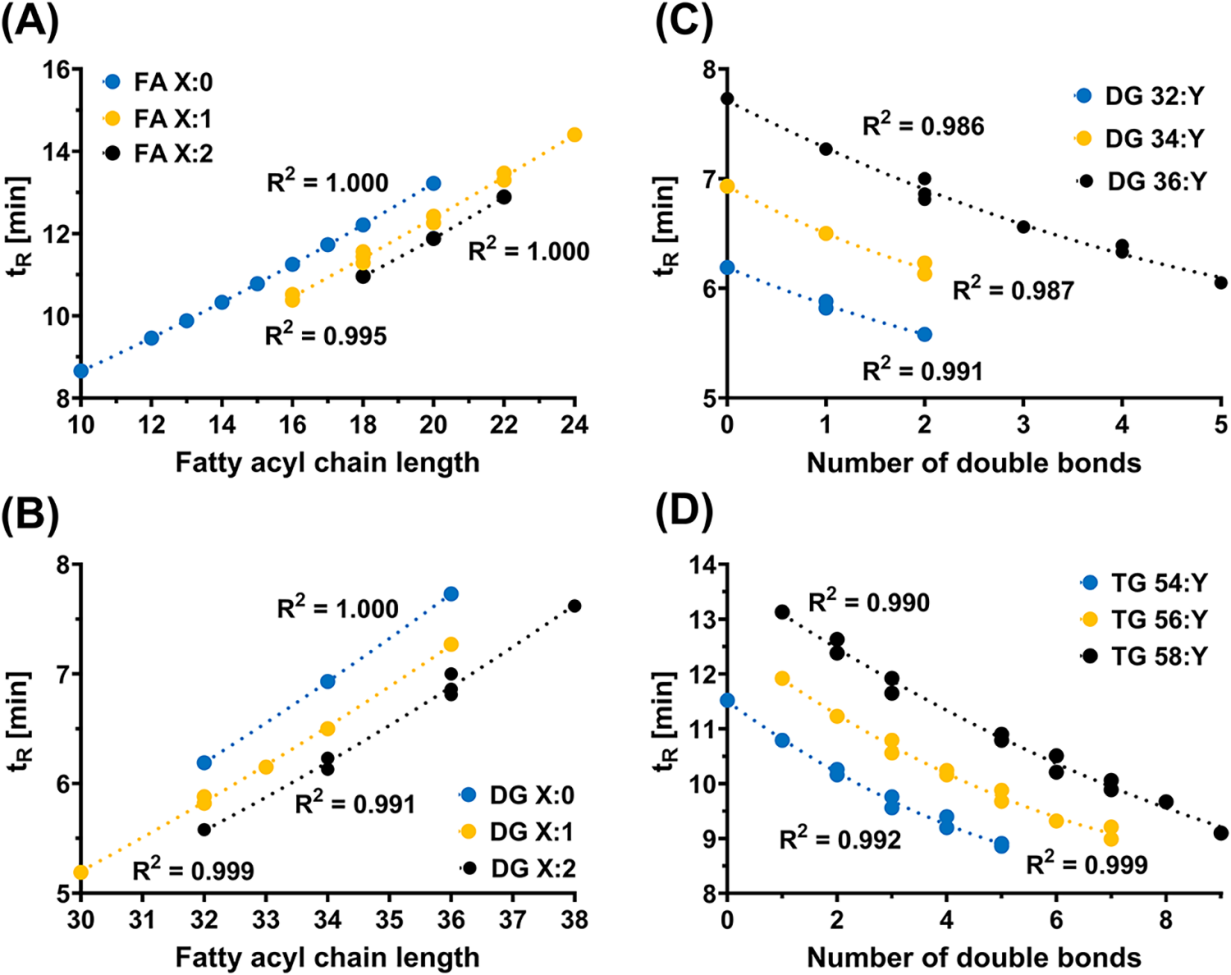
Retention behavior of various lipid species within lipid classes, illustrating polynomial dependencies of retention times on the fatty acyl chain length for (A) FA and (B) DG, and the number of double bonds for (C) DG and (D) TG.

**Table 1:** Comparison of the Number of Identified Less Polar Lipid Species in Human Plasma with Published Methods

	reversed-phase chromatography techniques				various techniques	
	current method	method 1 ^31^	method 2 ^32^	method 3 ^27^	method 4 ^37^	method 5 ^38^
FA	32	14	31	8	11	31
MG	6	4	not reported	5	not reported	not reported
DG	29^a^	20	12	40	23	55
TG	102	94	139	not reported	18	18
SE	15	13	21	not reported	16	22
ST	1	1	1	1	1	14^b^
total	156	146	204	54	69	126

a Sum of *sn*-1,2 and *sn*-1,3 isomers.

b Data represent a compilation from multiple targeted analytical strategies, such as gas chromatography–mass spectrometry or specialized method for sterol analysis.

Moreover, it is important to mention that our reported number of TG species is not final, as it is based on the number of chromatographic peaks and does not take into account the multiple possible isomeric compositions that may coelute within individual peaks. Although we report 29 DG species, this number is lower than that reported by Peterka et al.^27^ (40), likely due to differences in the derivatization strategy and method sensitivity. It is also lower than the 55 DG species reported by Quehenberger et al.^38^ (55), whose comprehensive approach allows for the distinction of *sn*-1,2 and *sn*-1,3 positional isomers, which is not achieved by our fast method. For FA, our method is consistent with the results reported by other approaches, further highlighting its advantage in separating isomers differing in the DB position. The greatest advantage of our method remains its ability to analyze all less polar lipid classes in a single ionization polarity mode, making the workflow less time-consuming and environmentally friendly (thanks to the use of CO_2_ and less amount of organic solvents) compared to methods requiring polarity switching or multiple analytical runs.

### Method Validation

The developed RP-UHPSFC/MS/MS method was validated using pooled human plasma spiked with ISmix and processed through a full derivatization workflow. The following validation parameters were evaluated in accordance with standard bioanalytical guidelines: linearity, limit of detection (LOD), limit of quantification (LOQ), instrumental precision, precision, accuracy, selectivity, carry-over, matrix effect, and recovery rate. Calibration curves were constructed by spiking pooled plasma with the ISmix before Folch extraction at multiple concentration levels, followed by derivatization and the final extraction into Hex (Figure S10). Each concentration point was prepared in triplicate and measured once. The resulting linear ranges, LOD, and LOQ for all IS are summarized in Table S5A. Carry-over was evaluated by injecting a blank solvent sample immediately after the highest calibration point. The signal in the blank was required to remain below 20% of the LOQ signal. Instrumental precision was determined by ten replicate injections of the derivatized pooled plasma sample spiked with ISmix before Folch extraction at the medium concentration level (ML). Repeatability (intraday precision) was evaluated using six independently prepared plasma extracts spiked with ISmix (in ML) before Folch extraction. The relative standard deviation (RSD) for both parameters was required to be ≤15%. Accuracy was validated under the same conditions by calculating concentrations from the calibration curves and comparing them with the theoretical spiked values. Quantification error was required to remain within ±20%. For the evaluation of selectivity, matrix effect, and recovery rate, individual plasma samples from four randomly selected females and four males and one pooled sample were analyzed. Selectivity was evaluated by analyzing each sample set with and without the addition of the ISmix at ML, after extraction into Hex. The intensity of interferences in the pure matrix was required not to exceed 20% of the IS signal intensity. The matrix effect was investigated by comparing the response of the sample set spiked with the ISmix at ML after extraction into Hex to the response of the neat ISmix solution at the same concentration. The matrix factor (MF) was calculated for each lipid class as the ratio of the peak area in the matrix to the peak area in the solvent. Only nonderivatized lipid classes were included in this experiment, and the %RSD of MFs across samples was required to be ≤ 15%. Recovery was assessed for nonderivatized lipid classes by comparing the signal from samples spiked before Folch extraction to those spiked after extraction into Hex. An overview of accuracy, precision (instrument precision and repeatability), selectivity, carry-over, matrix effect, and recovery rate results is shown in Table S5B. Almost all IS form nonpolar lipid classes included in this study successfully passed all evaluated validation parameters and can therefore be considered suitable for quantitative analysis. The only exception was the FA class, which showed limited quantification accuracy, with quantification errors exceeding 20%. Although FA standards fulfilled all of the other validation criteria, they cannot be reliably used for the accurate quantification of this lipid class.

### Quantitation of Lipids in NIST SRM 1950 Plasma

The validated RP-UHPSFC/MS/MS method was applied for the quantitation of less polar lipids in NIST SRM 1950 human plasma spiked with ISmix before Folch extraction. Unlike HILIC-based approaches, where lipids are separated by class, RP-based separations are primarily driven by the fatty acyl chain length and the degree of unsaturation. This principle leads to significant retention time differences among individual quantified lipid species and a single class-specific IS, thereby challenging robust correction for matrix effects and ionization suppression. To address this, our strategy employs multiple internal standards per lipid class (except for ST) to better model the behavior of endogenous species across the chromatographic gradient (full list in Table S2). This approach balances the need for analytical accuracy against the increased cost and complexity associated with a larger IS panel.

SE exhibit various ionization efficiencies in MS depending on their structure (i.e., DB number and fatty acyl chain length), and therefore, the use of specific response factors (RF) is essential for their accurate quantification.^39,40^ In our study, RFs for 17 cholesteryl ester (CE) standards were determined by calculating the ratio of their calibration curve slopes relative to a reference compound, CE 20:0 (RF = 1), as described by the following equation: RF­(CE X:Y) = *a*(CE 20:0)/*a*(CE X:Y), where *a* represents the slope of the calibration curve. The resulting RF values (Table S6) show predictable trends based on the fatty acyl chain length and unsaturation level (Figure S11). An additional correction was also applied to account for the different ionization behavior of the deuterated IS, CE 16:0-d7. The profound impact of this correction is illustrated in Figure 4, which compares uncorrected data, RF-corrected data, and literature values, clearly demonstrating that RFs are indispensable for the accurate quantification of the SE class.

**Figure 4:**
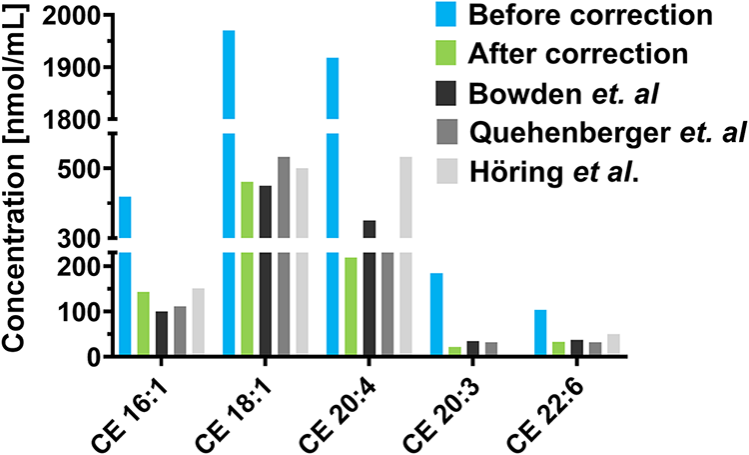
Effect of the application of response factor correction on the concentrations of selected sterol esters.

The final quantitative results for lipid species in NIST SRM 1950 plasma, calculated using all relevant IS and the application of response factors, are presented in Tables S7 and S8. The determined lipid concentrations were compared with results from our previously published HILIC-like UHPSFC/MS^30^ method and with data reported in other studies.^37,38,40,41^ Since the exact fatty acyl composition of many lipid species in the literature is unknown, comparisons were based on the summed concentrations of isomeric species. In general, our results showed good agreement with the literature data, with only minor deviations. These discrepancies can be attributed to inherent differences in extraction protocols, IS compositions, analytical approaches, instrumentation, and data processing workflows between studies. Although the IS for FA did not meet the accuracy criterion during method validation, other parameters, such as the precision and selectivity, remained within acceptable limits. This suggests that the method can still provide robust relative quantification for this class (Table S8), making the approach suitable for comparative analyses between distinct cohorts. The observed problem is most likely related to the instability of the derivatization products formed with BzCl. FA react with BzCl to form aromatic–FA mixed anhydrides, which are unstable and prone to disproportionation, leading to the formation of symmetrical aromatic–aromatic and FA–FA anhydrides.^42^ These side reactions negatively affect the reproducibility and quantification accuracy, making this derivatization approach unsuitable for reliable absolute FA quantitation. For this purpose, it is more appropriate to use derivatization reagents that do not produce unstable anhydrides. Alternative strategies may include the ester formation with p-dimethylaminophenacyl bromide,^43^ hydrazide derivatives with dansylhydrazine,^44^ or amides using reagents such as N2,N2,N4,N4-tetramethyl-6-(4-(piperazin-1ylsulfonyl)­phenyl)­1,3,5-triazine-2,4-diamine^45^ or 4-amino-1-benzylpiperidine,^46^ which offer an improved stability of derivatives and are more suitable for accurate FA quantification.

The successful quantitation of the other five lipid classes, coupled with the ability to obtain reliable relative data for the FA class, confirms the robustness of our integrated workflow. Ultimately, the minor discrepancies observed between our data and the literature values are not unexpected. Such variations are a common feature in lipidomics and underscore the ongoing need for greater standardization in analytical and data processing protocols in the lipidomics field.^47^

## Conclusions

In this work, we developed and validated a powerful RP-UHPSFC/MS/MS workflow for the detailed quantitative analysis of less polar lipids. By taking advantage of the unique properties of supercritical fluid chromatography, specifically its high affinity for less polar analytes and its capacity for rapid separations, our method successfully addresses a primary challenge in lipidomics. This challenge is the robust separation of structurally similar and isomeric species. The strategic combination of this technique with chemical derivatization and a high-resolution, dual-column setup resulted in a high-throughput platform capable of profiling six major lipid classes in a single 18-min analytical run. Our rigorous validation process confirms the method’s fitness for quantitative applications. It is important to note that correcting for structure-dependent ionization efficiencies via response factors is essential for the accurate quantification of certain classes such as sterol esters. The final concentrations determined for the NIST SRM 1950 plasma align well with established literature values, confirming the overall accuracy of our workflow. The minor discrepancies observed between data sets, however, are representative of a field-wide challenge, and they emphasize the continuing need for greater standardization in lipidomic protocols. For fatty acids, we recognize that derivatization with benzoyl chloride proceeds through mixed anhydrides that are unstable and prone to disproportionation, which likely contributed to the reduced performance observed during method validation. This limits the applicability for absolute quantitation of FA. In contrast, the method remains highly robust and reproducible for the other five lipid classes analyzed. This method also has the potential to be extended to other lipid classes, such as oxysterols or fatty acid esters of hydroxy fatty acids (FAhFAs), although further optimization of the extraction step prior to derivatization would be required, as these are minor lipids in human plasma. Moreover, benzoyl chloride derivatization can also be applied to more polar lipid subclasses containing free −OH or –NH_2_ functional groups when combined with suitable alternative chromatographic methods.

## Supplementary Material





## Notes

Supplementary Figures: optimization diagrams, example of MS/MS spectra, retention behaviors of lipids, calibration curves, and dependences of response factors. (PDF)
Supplementary Tables: composition of StdMix and ISmix, list of identified lipids and their fragments, comparison with other methods, validation results, response factors, and quantitation results. (XLSX)

## References

[1] Fahy E., Subramaniam S., Brown H. A., Glass C. K., Merrill A. H., Murphy R. C., Raetz C. R. H., Russell D. W., Seyama Y., Shaw W., Shimizu T., Spener F., Van Meer G., VanNieuwenhze M. S., White S. H., Witztum J. L., Dennis E. A. (2005). A Comprehensive
Classification System for Lipids. J. Lipid Res..

[2] Holčapek M., Liebisch G., Ekroos K. (2018). Lipidomic Analysis. Anal. Chem..

[3] Wang J., Wang C., Han X. (2019). Tutorial on
Lipidomics. Anal. Chim. Acta.

[4] Holčapek M., Ovčačíková M., Lísa M., Cífková E., Hájek T. (2015). Continuous
Comprehensive Two-Dimensional Liquid Chromatography–Electrospray
Ionization Mass Spectrometry of Complex Lipidomic Samples. Anal. Bioanal. Chem..

[5] Han X., Gross R. W. (2003). Global Analyses
of Cellular Lipidomes Directly from
Crude Extracts of Biological Samples by ESI Mass Spectrometry: A Bridge
to Lipidomics. J. Lipid Res..

[6] Han X., Yang K., Gross R. W. (2012). Multi-Dimensional
Mass Spectrometry-Based
Shotgun Lipidomics and Novel Strategies for Lipidomic Analyses. Mass Spectrom. Rev..

[7] Cajka T., Fiehn O. (2014). Comprehensive Analysis of Lipids in Biological Systems by Liquid
Chromatography-Mass Spectrometry. TrAC, Trends
Anal. Chem..

[8] Triebl A., Hartler J., Trötzmüller M., Köfeler H. C. (2017). Lipidomics:
Prospects from a Technological Perspective. Biochim. Biophys. Acta.

[9] Chipeaux C., de Person M., Burguet N., Billette de Villemeur T., Rose C., Belmatoug N., Héron S., Le Van Kim C., Franco M., Moussa F. (2017). Optimization of Ultra-High
Pressure Liquid Chromatography – Tandem Mass Spectrometry Determination
in Plasma and Red Blood Cells of Four Sphingolipids and Their Evaluation
as Biomarker Candidates of Gaucher’s Disease. J. Chromatogr. A.

[10] Lerner R., Baker D., Schwitter C., Neuhaus S., Hauptmann T., Post J. M., Kramer S., Bindila L. (2023). Four-Dimensional Trapped
Ion Mobility Spectrometry Lipidomics for High Throughput Clinical
Profiling of Human Blood Samples. Nat. Commun..

[11] Peterka O., Maccelli A., Jirásko R., Vaňková Z., Idkowiak J., Hrstka R., Wolrab D., Holčapek M. (2024). HILIC/MS Quantitation
of Low-Abundant Phospholipids and Sphingolipids in Human Plasma and
Serum: Dysregulation in Pancreatic Cancer. Anal.
Chim. Acta.

[12] Medina J., van der Velpen V., Teav T., Guitton Y., Gallart-Ayala H., Ivanisevic J. (2020). Single-Step Extraction Coupled with Targeted Hilic-Ms/Ms
Approach for Comprehensive Analysis of Human Plasma Lipidome and Polar
Metabolome. Metabolites.

[13] Cífková E., Holčapek M., Lísa M., Vrána D., Melichar B., Študent V. (2015). Lipidomic Differentiation between
Human Kidney Tumors and Surrounding Normal Tissues Using HILIC-HPLC/ESI-MS
and Multivariate Data Analysis. J. Chromatogr.
B.

[14] Zhang Z., Singh M., Kindt A., Wegrzyn A. B., Pearson M. J., Ali A., Harms A. C., Baker P., Hankemeier T. (2023). Development
of a Targeted Hydrophilic Interaction Liquid Chromatography-Tandem
Mass Spectrometry Based Lipidomics Platform Applied to a Coronavirus
Disease Severity Study. J. Chromatogr. A.

[15] Bamba T., Lee J. W., Matsubara A., Fukusaki E. (2012). Metabolic Profiling
of Lipids by Supercritical Fluid Chromatography/Mass Spectrometry. J. Chromatogr. A.

[16] Chocholoušková M., Torta F. (2025). Fast and Comprehensive
Lipidomic Analysis Using Supercritical Fluid
Chromatography Coupled with Low and High Resolution Mass Spectrometry. J. Chromatogr. A.

[17] Lísa M., Holčapek M. (2015). High-Throughput and Comprehensive Lipidomic Analysis
Using Ultrahigh-Performance Supercritical Fluid Chromatography-Mass
Spectrometry. Anal. Chem..

[18] Wolrab D., Jirásko R., Cífková E., Höring M., Mei D., Chocholoušková M., Peterka O., Idkowiak J., Hrnčiarová T., Kuchař L., Ahrends R., Brumarová R., Friedecký D., Vivo-Truyols G., Škrha P., Škrha J., Kučera R., Melichar B., Liebisch G., Burkhardt R., Wenk M. R., Cazenave-Gassiot A., Karásek P., Novotný I., Greplová K., Hrstka R., Holčapek M. (2022). Lipidomic
Profiling of Human Serum Enables Detection of Pancreatic Cancer. Nat. Commun..

[19] Peterka O., Jirásko R., Chocholoušková M., Kuchař L., Wolrab D., Hájek R., Vrána D., Strouhal O., Melichar B., Holčapek M. (2020). Lipidomic
Characterization of Exosomes Isolated from Human Plasma Using Various
Mass Spectrometry Techniques. Biochim. Biophys.
Acta.

[20] Chollet C., Boutet-Mercey S., Laboureur L., Rincon C., Méjean M., Jouhet J., Fenaille F., Colsch B., Touboul D. (2019). Supercritical
Fluid Chromatography Coupled to Mass Spectrometry for Lipidomics. J. Mass Spectrom..

[21] Losacco G. L., Veuthey J. L., Guillarme D. (2019). Supercritical
Fluid Chromatography
– Mass Spectrometry: Recent Evolution and Current Trends. TrAC, Trends Anal. Chem..

[22] Guillarme D., Desfontaine V., Heinisch S., Veuthey J. L. (2018). What Are the Current
Solutions for Interfacing Supercritical Fluid Chromatography and Mass
Spectrometry?. J. Chromatogr. B.

[23] Laboureur L., Ollero M., Touboul D. (2015). Lipidomics by Supercritical Fluid
Chromatography. Int. J. Mol. Sci..

[24] Berkecz R., Lísa M., Holčapek M. (2017). Analysis of Oxylipins in Human Plasma:
Comparison of Ultrahigh-Performance Liquid Chromatography and Ultrahigh-Performance
Supercritical Fluid Chromatography Coupled to Mass Spectrometry. J. Chromatogr. A.

[25] Ubhayasekera S. J. K. A., Acharya S. R., Bergquist J. (2018). A Novel, Fast
and Sensitive Supercritical
Fluid Chromatography-Tandem Mass Spectrometry (SFC-MS/MS) Method for
Analysis of Arachidonic Acid Metabolites. Analyst.

[26] Wolrab D., Chocholoušková M., Jirásko R., Peterka O., Holčapek M. (2020). Validation
of Lipidomic Analysis
of Human Plasma and Serum by Supercritical Fluid Chromatography–Mass
Spectrometry and Hydrophilic Interaction Liquid Chromatography–Mass
Spectrometry. Anal. Bioanal. Chem..

[27] Peterka O., Jirásko R., Vaňková Z., Chocholoušková M., Wolrab D., Kulhánek J., Bureš F., Holčapek M. (2021). Simple and Reproducible Derivatization with Benzoyl
Chloride: Improvement of Sensitivity for Multiple Lipid Classes in
RP-UHPLC/MS. Anal. Chem..

[28] Pino L. K., Searle B. C., Bollinger J. G., Nunn B., MacLean B., MacCoss M. J. (2020). The Skyline Ecosystem:
Informatics for Quantitative
Mass Spectrometry Proteomics. Mass Spectrom.
Rev..

[29] Hájek R., Lísa M., Khalikova M., Jirásko R., Cífková E., Študent V., Vrána D., Opálka L., Vávrová K., Matzenauer M., Melichar B., Holčapek M. (2018). HILIC/ESI-MS
Determination of Gangliosides and Other Polar Lipid Classes in Renal
Cell Carcinoma and Surrounding Normal Tissues. Anal. Bioanal. Chem..

[30] Wolrab D., Peterka O., Chocholoušková M., Holčapek M. (2022). Ultrahigh-Performance Supercritical Fluid Chromatography/Mass
Spectrometry in the Lipidomic Analysis. TrAC,
Trends Anal. Chem..

[31] Vaňková Z., Peterka O., Chocholoušková M., Wolrab D., Jirásko R., Holčapek M. (2022). Retention
Dependences Support Highly Confident Identification of Lipid Species
in Human Plasma by Reversed-Phase UHPLC/MS. Anal. Bioanal. Chem..

[32] Ovčačíková M., Lísa M., Cífková E., Holčapek M. (2016). Retention
Behavior of Lipids in Reversed-Phase Ultrahigh-Performance Liquid
Chromatography-Electrospray Ionization Mass Spectrometry. J. Chromatogr. A.

[33] Folch J., Lees M., Stanley G. (1957). A Simple Method for
the Isolation
and Purification of Total Lipides from Animal Tissues. J. Biol. Chem..

[34] Bligh E. G., Dyer W. (1959). A Rapid Method of Total
Lipid Extraction And Purification. Can. J. Biochem.
Physiol..

[35] Matyash V., Liebisch G., Kurzchalia T. V., Shevchenko A., Schwudke D. (2008). Lipid Extraction by Methyl-Terf-Butyl
Ether for High-Throughput
Lipidomics. J. Lipid Res..

[36] Liebisch G., Fahy E., Aoki J., Dennis A. E., Durand T., Ejsing S. C., Fedorova M., Feussner I., Griffiths J. W., Köfeler H., Merrill H. A., Murphy C. R., O’Donnell B. V., Oskolkova O., Subramaniam S., Wakelam J. O. M., Spener F. (2020). Update on
LIPID MAPS Classification,
Nomenclature, and Shorthand Notation for MS-Derived Lipid Structures. J. Lipid Res..

[37] Bowden J. A., Heckert A., Ulmer C. Z., Jones C. M., Koelmel J. P., Abdullah L., Ahonen L., Alnouti Y., Armando A. M., Asara J. M., Bamba T., Barr J. R., Bergquist J., Borchers C. H., Brandsma J., Breitkopf S. B., Cajka T., Cazenave-Gassiot A., Checa A., Cinel M. A., Colas R. A., Cremers S., Dennis E. A., Evans J. E., Fauland A., Fiehn O., Gardner M. S., Garrett T. J., Gotlinger K. H., Han J., Huang Y., Neo A. H., Hyötyläinen T., Izumi Y., Jiang H., Jiang H., Jiang J., Kachman M., Kiyonami R., Klavins K., Klose C., Köfeler H. C., Kolmert J., Koal T., Koster G., Kuklenyik Z., Kurland I. J., Leadley M., Lin K., Maddipati K. R., McDougall D., Meikle P. J., Mellett N. A., Monnin C., Moseley M. A., Nandakumar R., Oresic M., Patterson R., Peake D., Pierce J. S., Post M., Postle A. D., Pugh R., Qiu Y., Quehenberger O., Ramrup P., Rees J., Rembiesa B., Reynaud D., Roth M. R., Sales S., Schuhmann K., Schwartzman M. L., Serhan C. N., Shevchenko A., Somerville S. E., St John-Williams L., Surma M. A., Takeda H., Thakare R., Thompson J. W., Torta F., Triebl A., Trötzmüller M., Ubhayasekera S. J. K., Vuckovic D., Weir J. M., Welti R., Wenk M. R., Wheelock C. E., Yao L., Yuan M., Zhao X. H., Zhou S. (2017). Harmonizing Lipidomics: NIST Interlaboratory Comparison Exercise
for Lipidomics Using SRM 1950-Metabolites in Frozen Human Plasma. J. Lipid Res..

[38] Quehenberger O., Armando A. M., Brown A. H., Milne S. B., Myers D. S., Merrill A. H., Bandyopadhyay S., Jones K. N., Kelly S., Shaner R. L., Sullards C. M., Wang E., Murphy R. C., Barkley R. M., Leiker T. J., Raetz C. R. H., Guan Z., Laird G. M., Six D. A., Russell D. W., McDonald J. G., Subramaniam S., Fahy E., Dennis E. A. (2010). Lipidomics Reveals
a Remarkable Diversity of Lipids in Human Plasma1. J. Lipid Res..

[39] Holčapek M., Lísa M., Jandera P., Kabátová N. (2005). Quantitation
of Triacylglycerols in Plant Oils Using HPLC with APCI-MS, Evaporative
Light-Scattering, and UV Detection. J. Sep.
Sci..

[40] Höring M., Ejsing C. S., Hermansson M., Liebisch G. (2019). Quantification of Cholesterol
and Cholesteryl Ester by Direct Flow Injection High-Resolution Fourier
Transform Mass Spectrometry Utilizing Species-Specific Response Factors. Anal. Chem..

[41] Ghorasaini M., Mohammed Y., Adamski J., Bettcher L., Bowden J. A., Cabruja M., Contrepois K., Ellenberger M., Gajera B., Haid M., Hornburg D., Hunter C., Jones C. M., Klein T., Mayboroda O., Mirzaian M., Moaddel R., Ferrucci L., Lovett J., Nazir K., Pearson M., Ubhi B. K., Raftery D., Riols F., Sayers R., Sijbrands E. J. G., Snyder M. P., Su B., Velagapudi V., Williams K. J., De Rijke Y. B., Giera M. (2021). Cross-Laboratory Standardization
of Preclinical Lipidomics Using Differential Mobility Spectrometry
and Multiple Reaction Monitoring. Anal. Chem..

[42] Trabelsi I., Essid K., Frikha M. H. (2017). Synthesis of Mixed Anhydrides of
Fatty Acids: Stability and Reactivity. Ind.
Crops Prod..

[43] Guo K., Li L. (2010). High-Performance
Isotope Labeling for Profiling Carboxylic Acid-Containing
Metabolites in Biofluids by Mass Spectrometry. Anal. Chem..

[44] Zhao S., Li L. (2018). Dansylhydrazine Isotope Labeling LC-MS for Comprehensive Carboxylic
Acid Submetabolome Profiling. Anal. Chem..

[45] Li S., Xiao Q., Sun J., Li Z., Zhang M., Tian Y., Zhang Z., Dong H., Jiao Y., Xu F., Zhang P. (2024). A New Chemical
Derivatization Reagent Sulfonyl Piperazinyl
for the Quantification of Fatty Acids Using LC-MS/MS. Talanta.

[46] Yang J., Wu Y., Zhao L., Li M., Guo W., Xu Y., Wang Y. (2025). Chemical Derivatization-Based Liquid Chromatography-Mass Spectrometry
Method for Fatty Acid Profiling in Biological Samples. J. Sep. Sci..

[47] Liebisch G., Ahrends R., Arita M., Arita M., Bowden J. A., Ejsing C. S., Griffiths W. J., Holcapek M., Köfeler H., Mitchell T. W., Wenk M., Ekroos K. (2019). Lipidomics Needs More
Standardization. Nat. Metab..

