# Supplementary material for: Comprehensive Quantitative Profiling of Less Polar Lipids in Human Plasma Using Validated Reversed-Phase UHPSFC/MS/MS
Source: Anal Chem. 2025 Nov 24;97(48):26501–10. doi: 10.1021/acs.analchem.5c04668 (PMC12874210; doi:10.1021/acs.analchem.5c04668)

**SUPPORTING INFORMATION**

# Comprehensive Quantitative Profiling of Less Polar Lipids in Human Plasma Using Validated Reversed-Phase UHPSFC/MS/MS

Zuzana Lásko[#], Veronika Šubrtová[#], Ondřej Peterka, Robert Jirásko, Michal Holčapek[*]

*Department of Analytical Chemistry, Faculty of Chemical Technology, University of Pardubice, Studentská 573, 53210 Pardubice, Czech Republic*

[*] Corresponding author: Michal Holčapek, Tel.: +420-466037087; Fax: +420-466037068; E-mail: Michal.Holcapek@upce.cz

Author contributions:

[#] Z.L. and V.Š. contributed equally to this work.

Content:

Number of figures: 10

**Table of Contents:**

**Figure S1:** Comparison of different derivatization reaction times and extraction protocols for the termination of reaction and the isolation of less polar lipid from derivatized pooled plasma.. 3
**Figure S2:** Influence of four different modifier solvents on the separation of a standard mixture. 4
**Figure S3:** Effect of the concentration of ammonium acetate and acetic acid in the modifier on the ionization efficiency of selected internal standards. 9
**Figure S4:** Influence of three different temperatures on the separation of a internal standard mixture. 10
**Figure S5:** Example of the separation of FA 18:1 and TG 54:3 isomers on a single column and on two columns connected in series. 11
**Figure S6:** Effect of the ion source temperature and desolvation temperature on the ionization of selected internal standards. 12
**Figure S7:** Example of MS/MS spectrum annotation for TG 54:5. 13
**Figure S8:** Retention behavior of various lipid species within lipid classes, illustrating polynomial dependencies of retention time on the fatty acyl chain length. 14
**Figure S9:** Retention behavior of various lipid species within lipid classes, illustrating polynomial dependencies of retention time on the number of double bonds. 15
**Figure S10:** Calibration curves of internal standards in spiked human plasma. 16
**Figure S11:** Dependences of response factors on fatty acyl chain length and on the number of double bonds. 17

**Figure S1:** Comparison of **(A)** different derivatization reaction times and **(B)** three extraction protocols for the termination of reaction and the isolation of less polar lipid from derivatized pooled plasma spiked with ISmix. Data points represent the mean ± SD of three independent replicates.

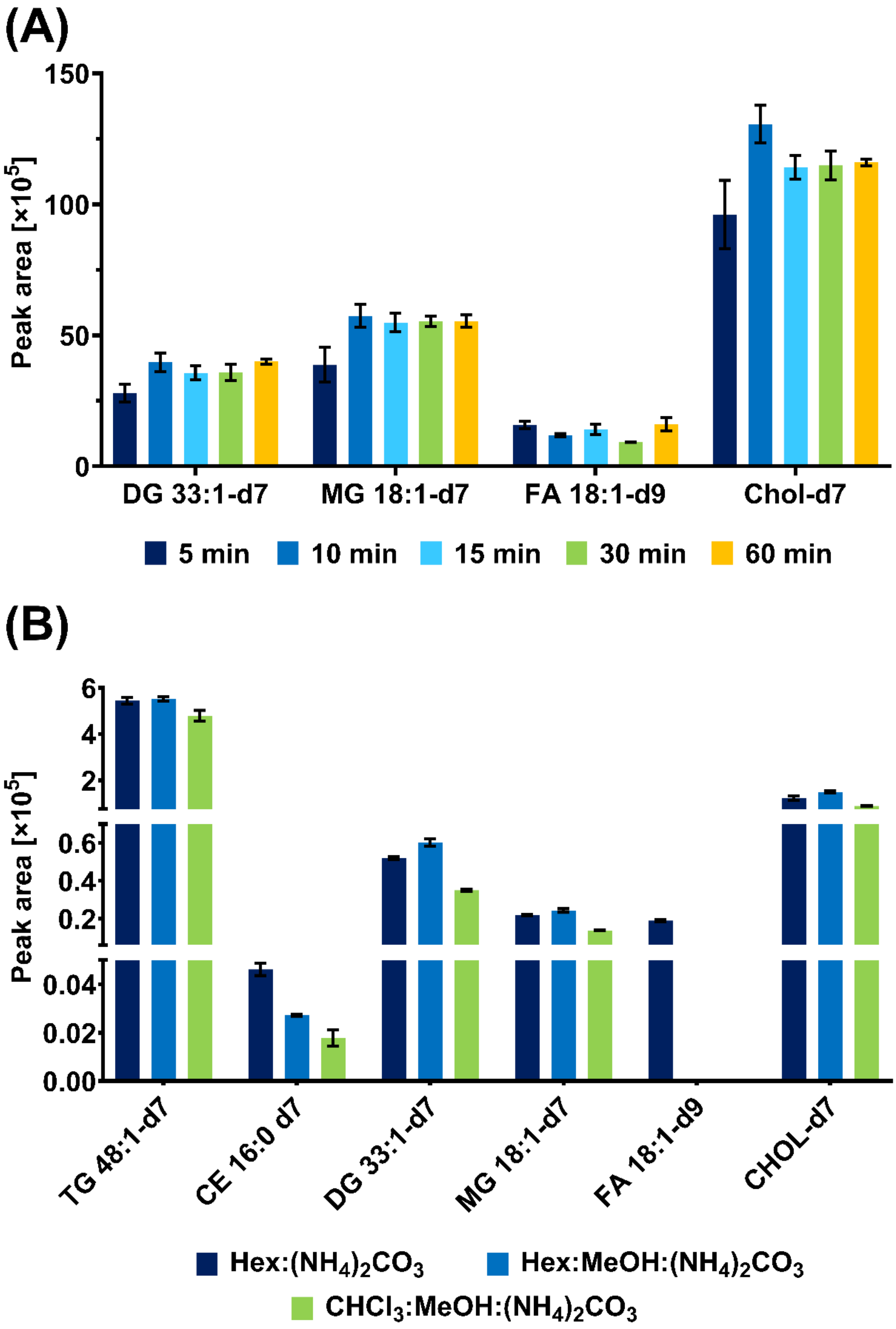

**Figure S2:** Influence of four different modifier solvents on the separation of a standard mixture containing **(A)** fatty acids, **(B)** monoacylglycerols, **(C)** diacylglycerols, **(D)** triacylglycerols, and **(E)** cholesteryl esters. The selected modifier is highlighted in green.

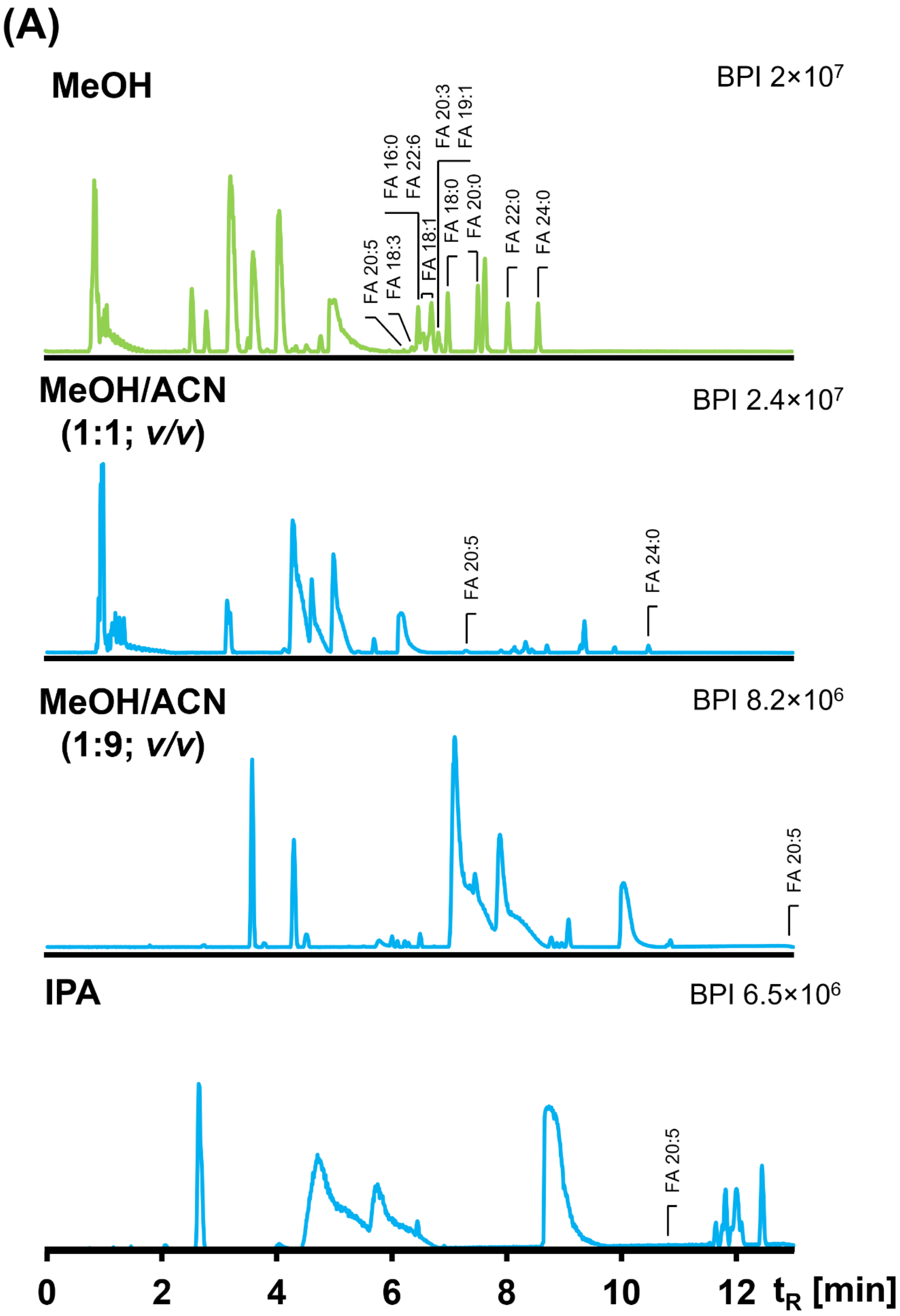

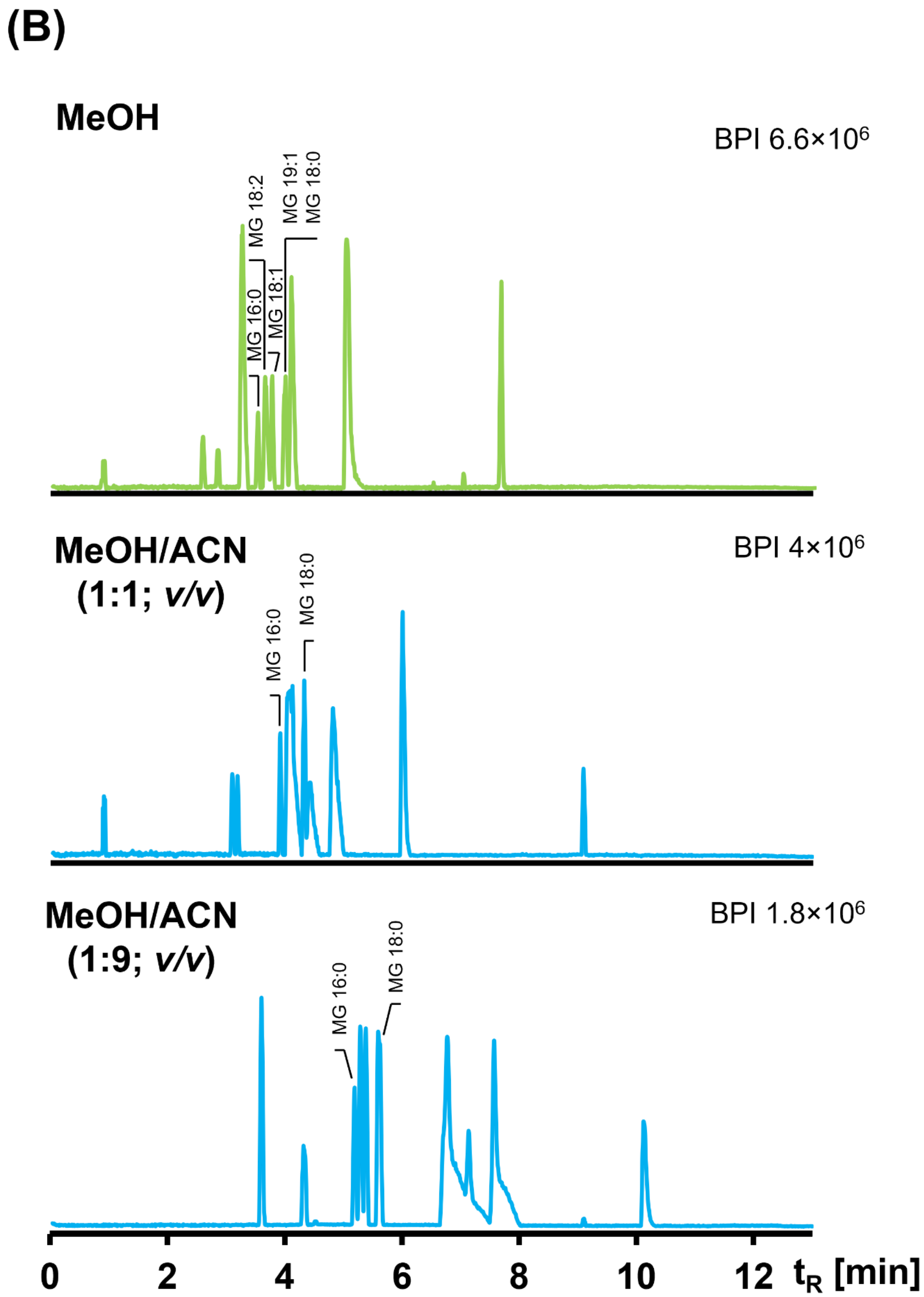
(B)
MeOH
BPI 6.6×10^6
MG 18:2
MG 16:0
MG 18:1
MG 19:1
MG 18:0
MeOH/ACN
(1:1; v/v)
BPI 4×10^6
MG 16:0
MG 18:0
MeOH/ACN
(1:9; v/v)
BPI 1.8×10^6
MG 16:0
MG 18:0
0
2
4
6
8
10
12
tR [min]

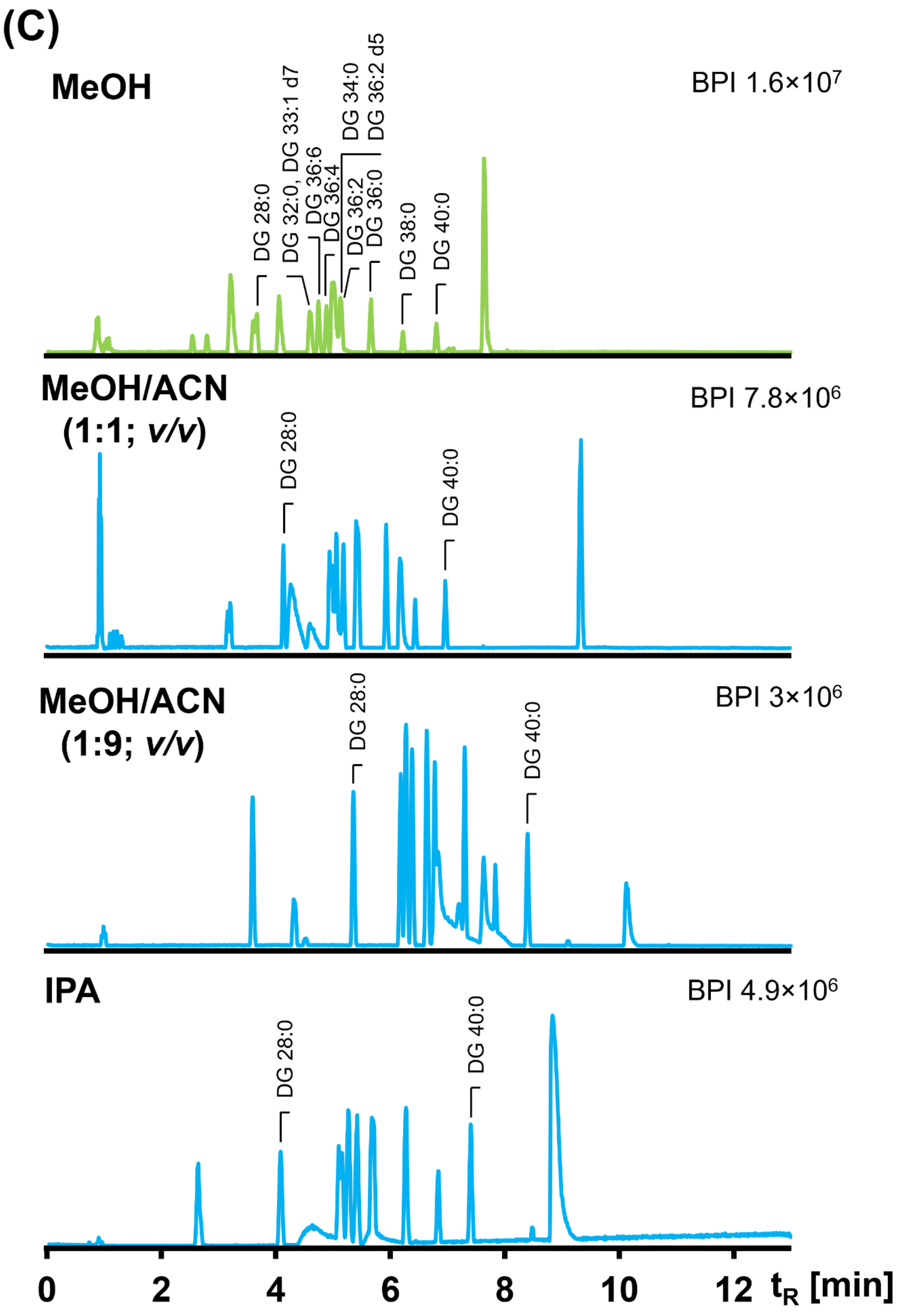

(C)
MeOH
BPI 1.6×10^7
DG 28:0
DG 32:0, DG 33:1 d7
DG 36:6
DG 36:4
DG 34:0
DG 36:2 d5
DG 36:2
DG 36:0
DG 38:0
DG 40:0
MeOH/ACN (1:1; v/v)
BPI 7.8×10^6
DG 28:0
DG 40:0
MeOH/ACN (1:9; v/v)
BPI 3×10^6
DG 28:0
DG 40:0
IPA
BPI 4.9×10^6
DG 28:0
DG 40:0
0
2
4
6
8
10
12
tR [min]

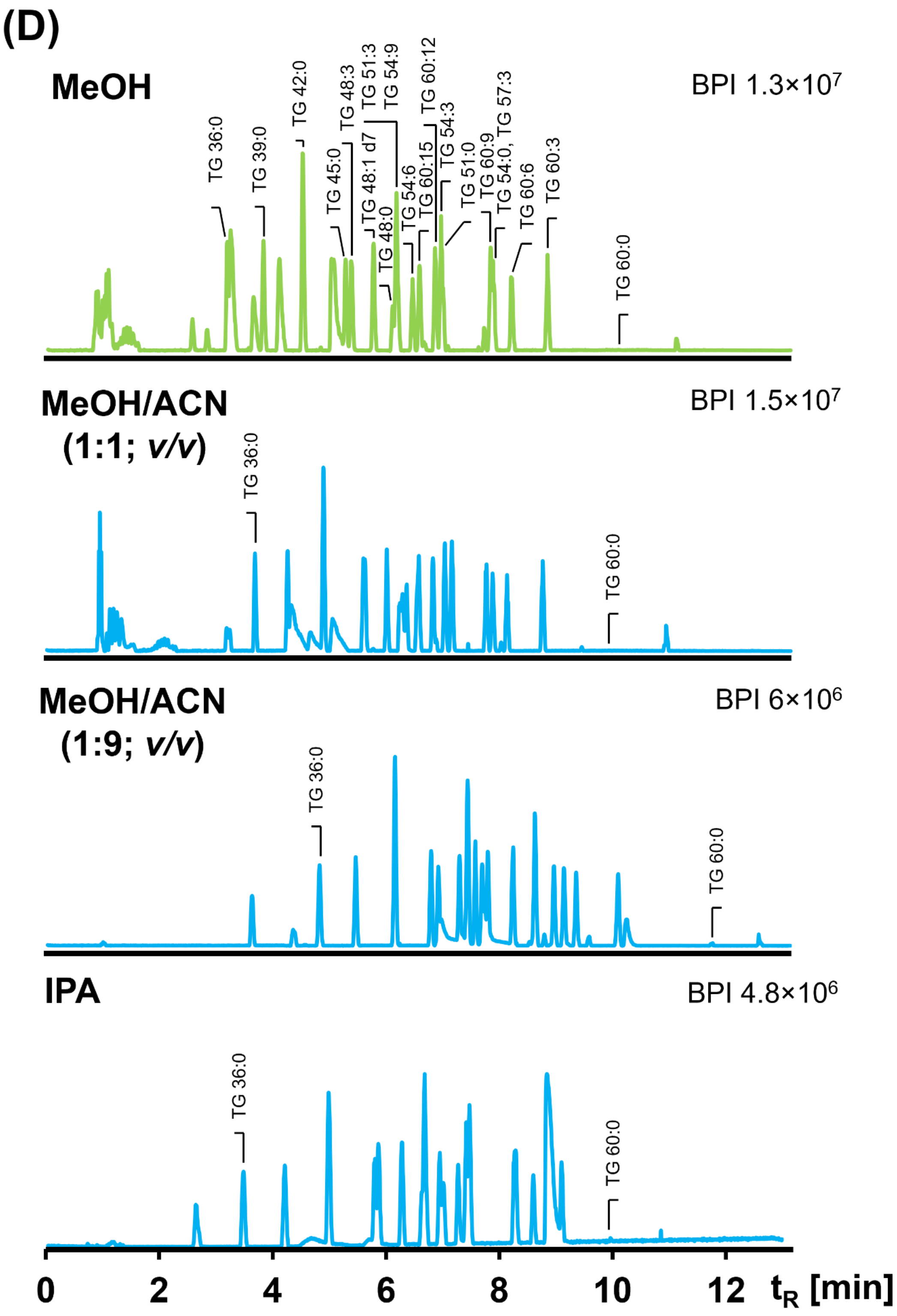
(D)
MeOH
BPI 1.3×10⁷
TG 36:0
TG 39:0
TG 42:0
TG 45:0
TG 48:3
TG 48:1 d7
TG 51:3
TG 54:9
TG 48:0
TG 54:6
TG 60:15
TG 60:12
TG 54:3
TG 51:0
TG 60:9
TG 54:0, TG 57:3
TG 60:6
TG 60:3
TG 60:0
MeOH/ACN
(1:1; v/v)
BPI 1.5×10⁷
TG 36:0
TG 60:0
MeOH/ACN
(1:9; v/v)
BPI 6×10⁶
TG 36:0
TG 60:0
IPA
BPI 4.8×10⁶
TG 36:0
TG 60:0
0
2
4
6
8
10
12
tR [min]

(E)

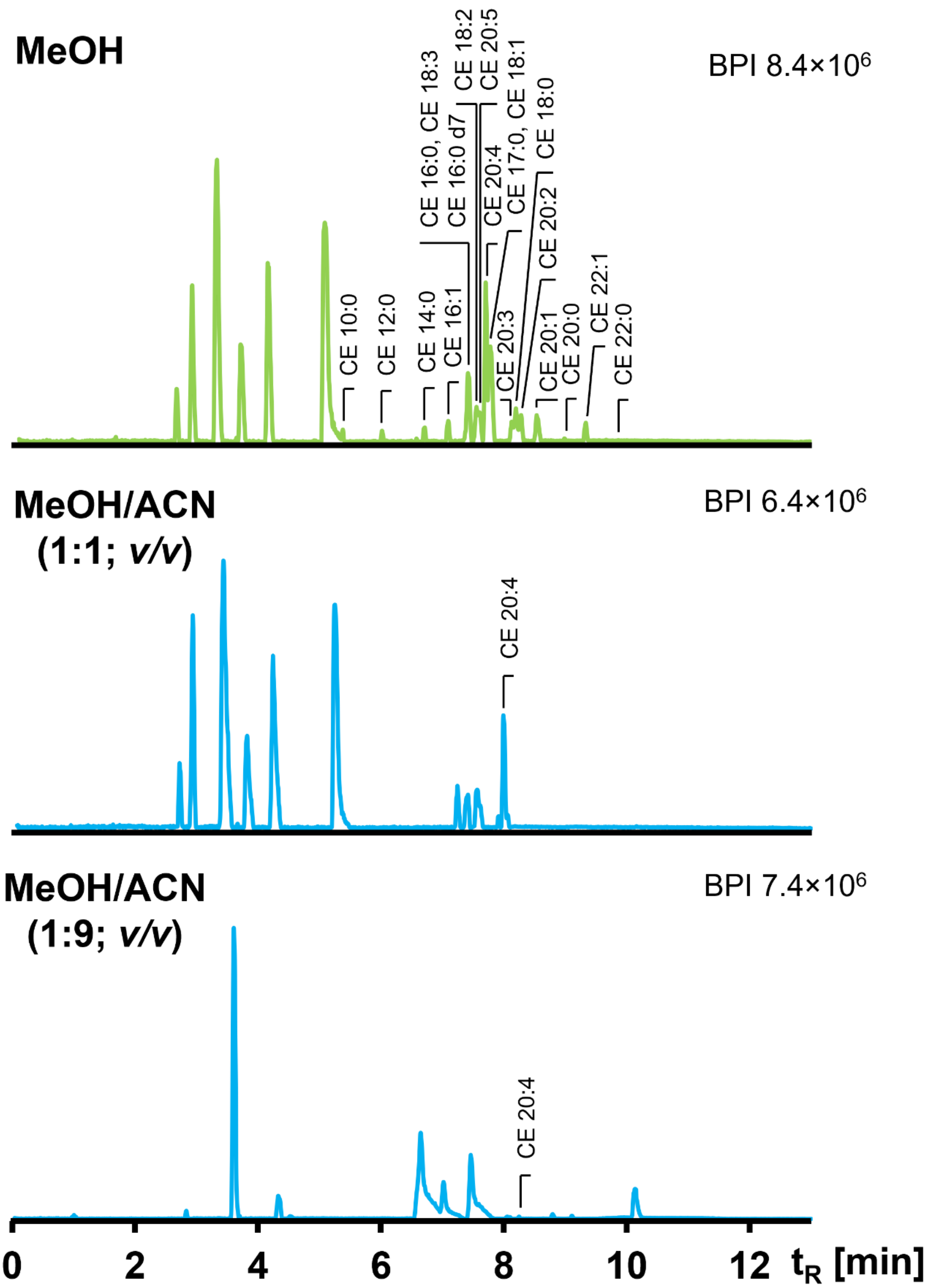
MeOH
BPI 8.4×10^6
CE 10:0
CE 12:0
CE 14:0
CE 16:1
CE 16:0, CE 18:3
CE 16:0 d7
CE 18:2
CE 20:5
CE 20:4
CE 20:3
CE 17:0, CE 18:1
CE 18:0
CE 20:2
CE 20:1
CE 20:0
CE 22:1
CE 22:0
MeOH/ACN
(1:1; v/v)
BPI 6.4×10^6
CE 20:4
MeOH/ACN
(1:9; v/v)
BPI 7.4×10^6
CE 20:4
0
2
4
6
8
10
12
tR [min]

**Figure S3:** Effect of the concentration of **(A)** ammonium acetate (AmAc) and **(B)** acetic acid in the modifier on the ionization efficiency of selected internal standards. Data are presented as mean value ± SD from three independent experiments.

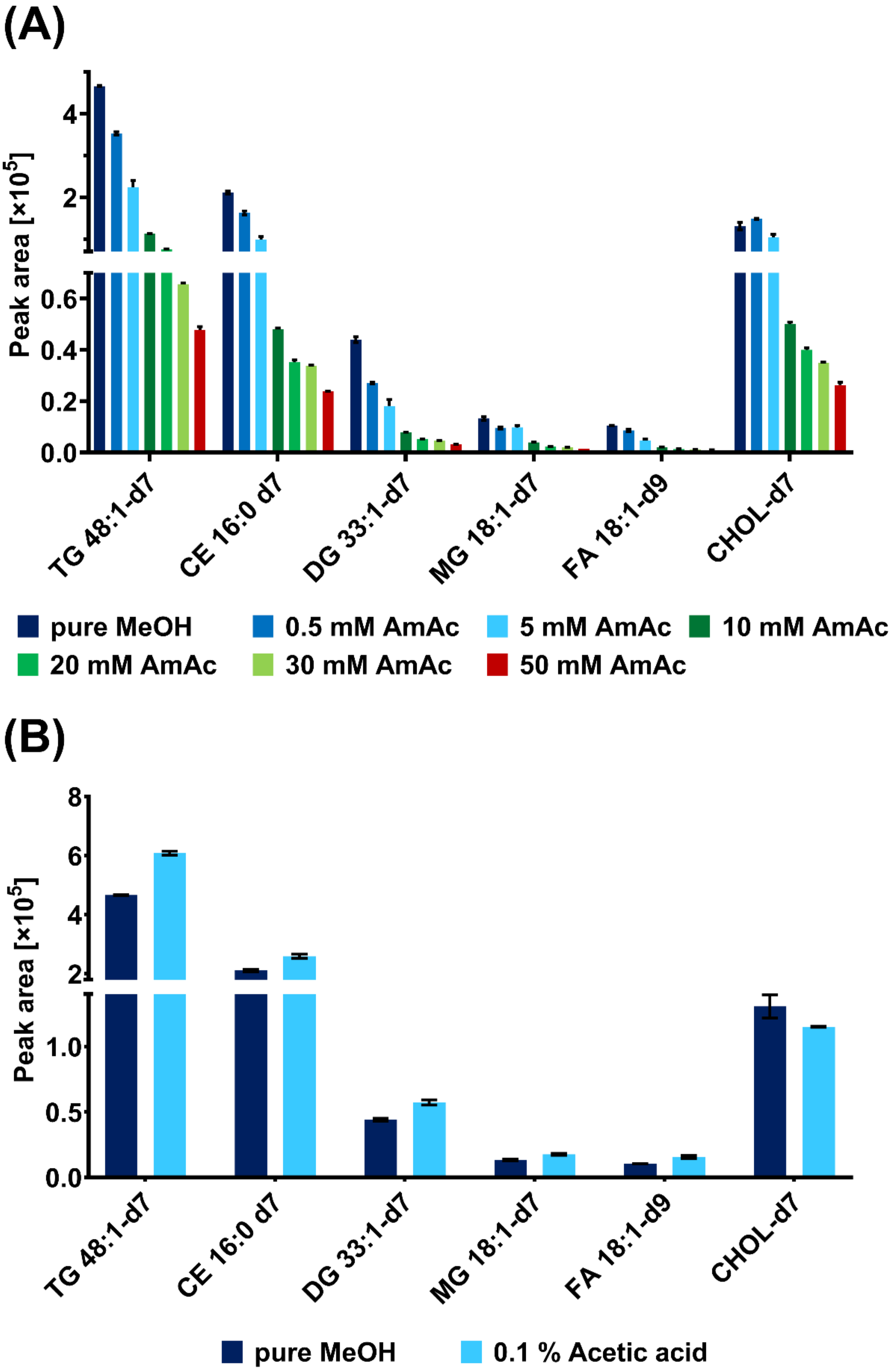

**Figure S4:** Influence of three different temperatures **(A)** 40 °C, **(B)** 50 °C, and **(C)** 60 °C on the separation of a internal standard mixture. The selected conditions are highlighted in green.

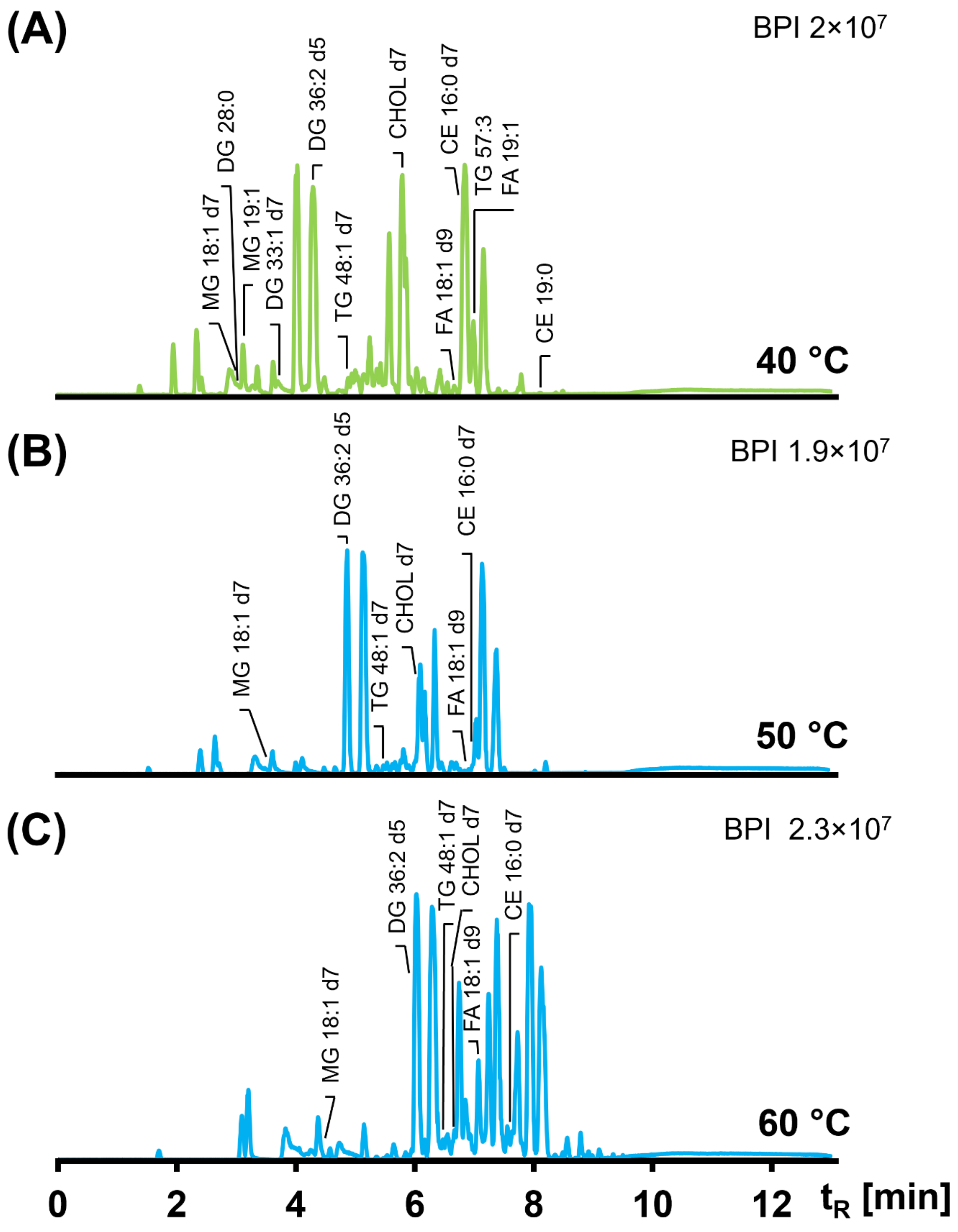

**Figure S5:** Example of the separation of FA 18:1 isomers **(A)** on a single column (150 × 3.0 mm; 1.8 µm, Waters) and **(B)** on two columns connected in series (100 × 3.0 mm; 1.8 µm and 150 × 3.0 mm; 1.8 µm, Waters), and TG 54:3 isomers **(C)** on a single column (150 × 3.0 mm; 1.8 µm, Waters) and **(D)** on two columns connected in series (100 × 3.0 mm; 1.8 µm and 150 × 3.0 mm; 1.8 µm, Waters).

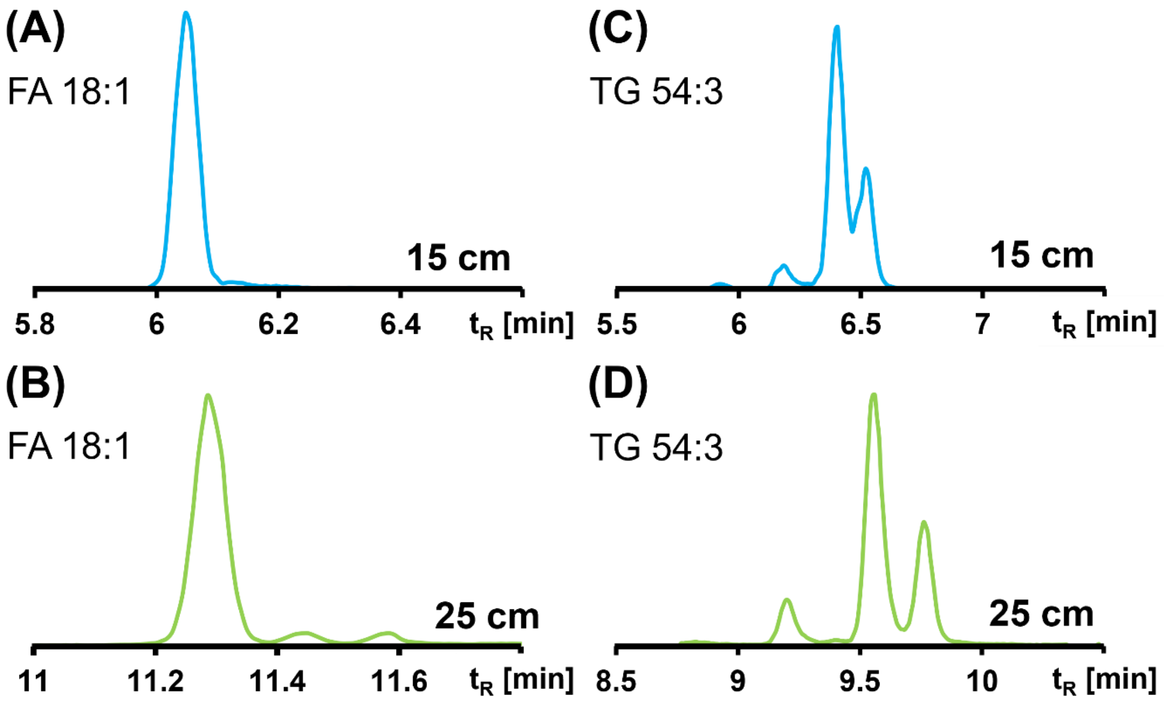

**Figure S6:** Effect of the **(A)** ion source temperature and **(B)** desolvation temperature on the ionization of selected internal standards. Data are presented as mean value ± SD from three independent experiments.

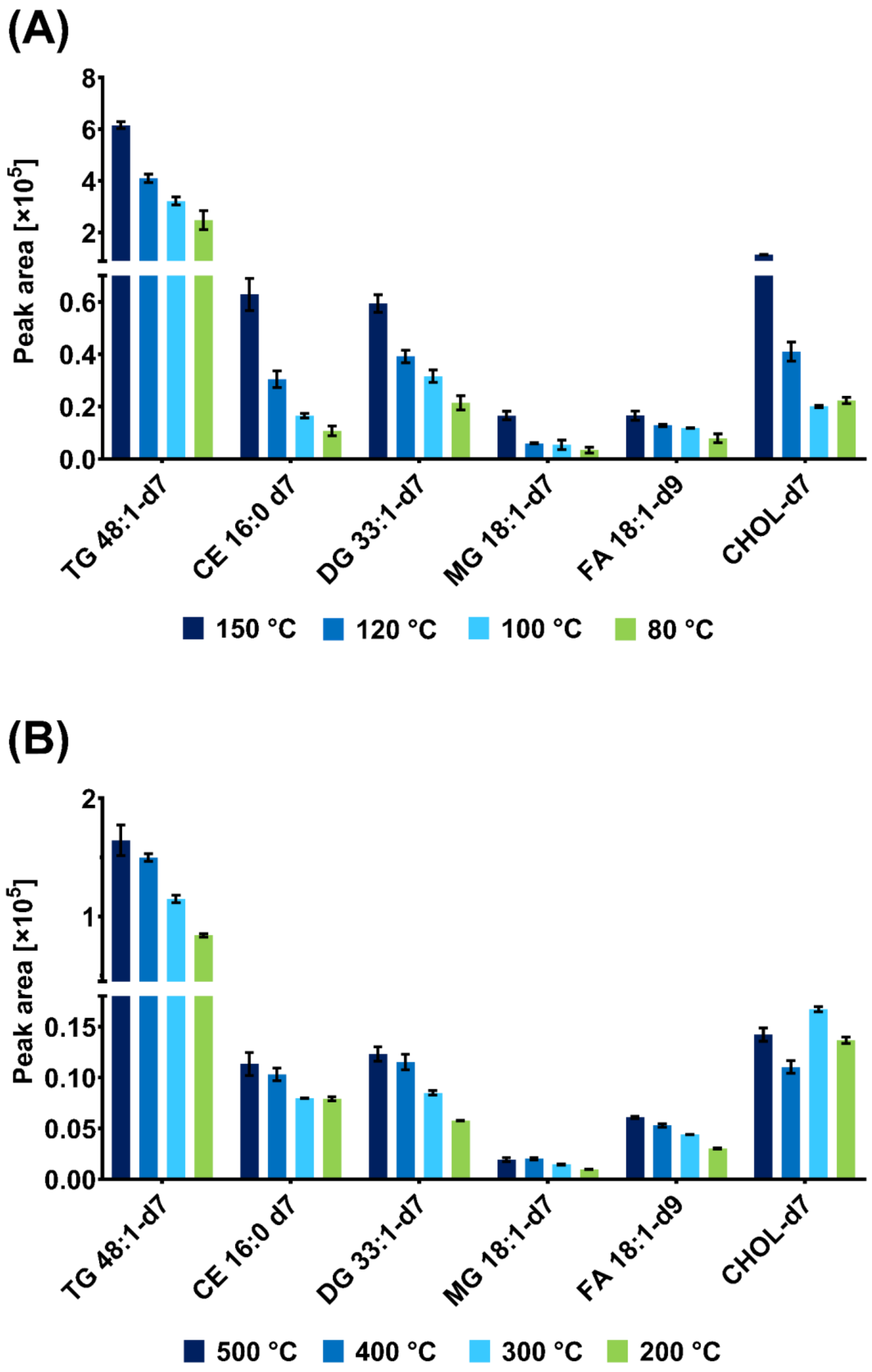

**Figure S7:** Example of MS/MS spectrum annotation for TG 54:5, illustrating characteristic fragment ions resulting from the neutral loss of individual fatty acids from the $[M+NH_4]^+$ precursor ion. NL … neutral loss.

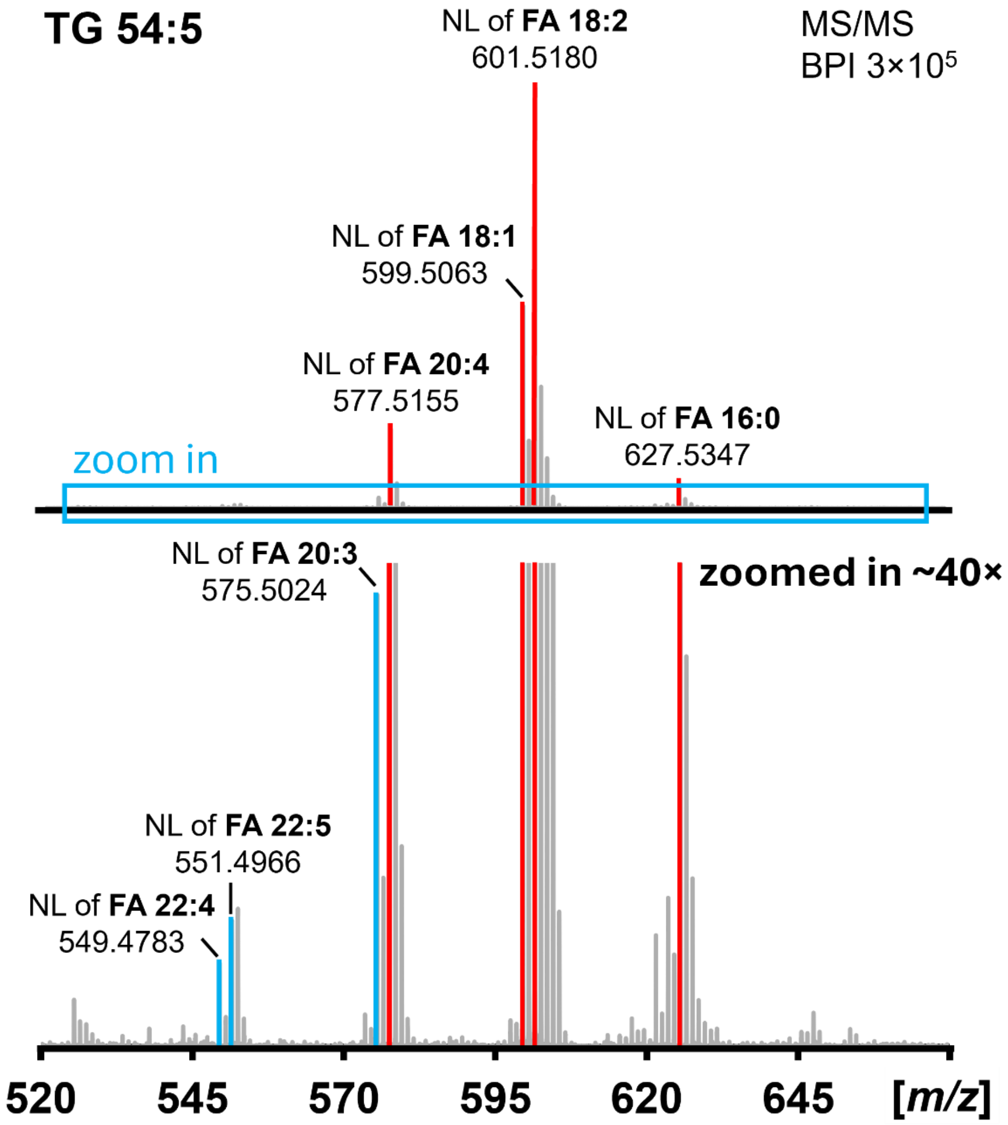

**Figure S8:** Retention behavior of various lipid species within lipid classes, illustrating polynomial dependencies of retention time on the fatty acyl chain length. **(A)** MG X:0; **(B)** TG X:0, TG X:1, and TG X:2; **(C)** TG X:3, TG X:4, and TG X:5; **(D)** SE 27:1/X:0. The variable X denotes the total number of carbon atoms in the lipid molecule, while the number after the colon indicates the number of double bonds.

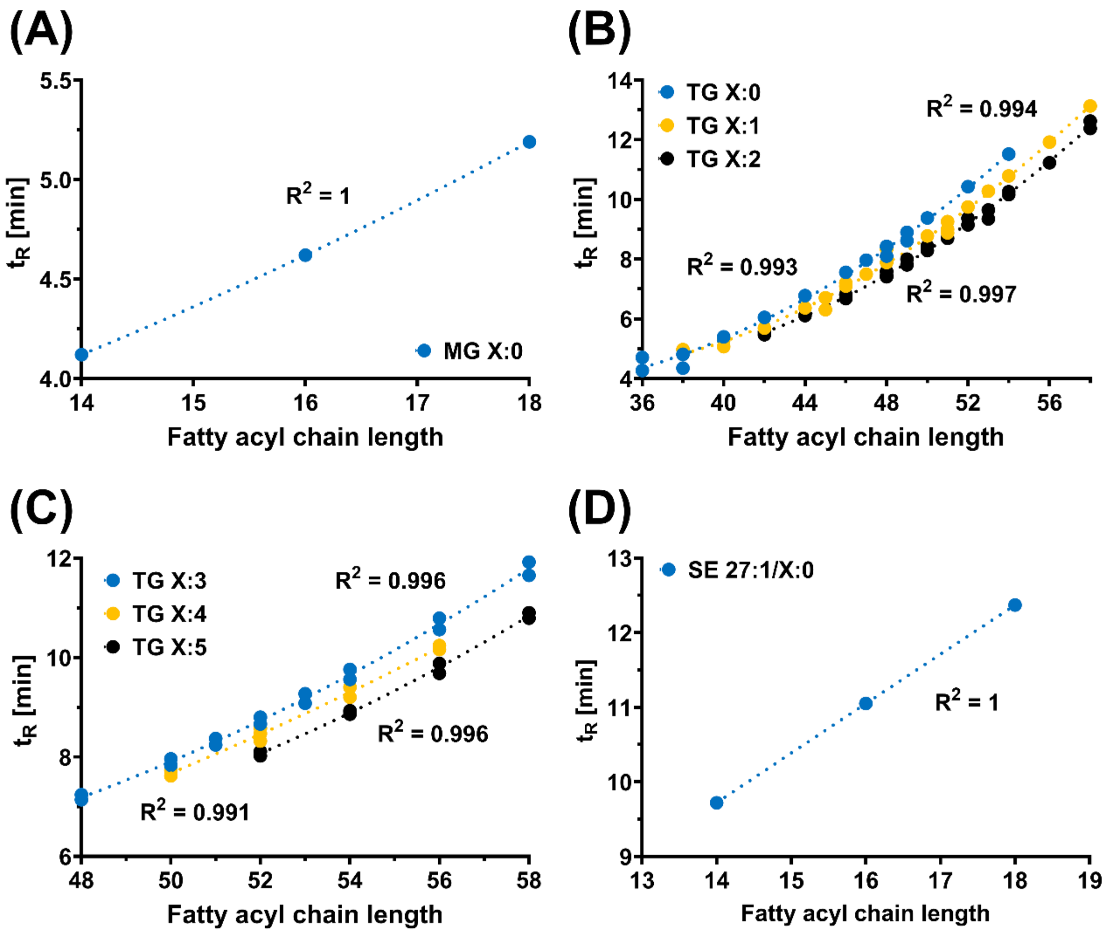

**Figure S9:** Retention behavior of various lipid species within lipid classes, illustrating polynomial dependencies of retention time on the number of double bonds. **(A)** FA 18:Y, FA 20:Y, and FA 22:Y; **(B)** MG 18:Y; **(C)** TG 42:Y, 44:Y, and 46:Y; **(D)** TG 48:Y, 50:Y, and TG 52:Y; **(E)** TG 49:Y, TG 51:Y, and TG 53:Y; **(F)** SE 27:1/18:Y and SE 27:1/20:Y. The number of carbon atoms is indicated before the colon, and Y denotes the number of double bond(s) in the molecule.

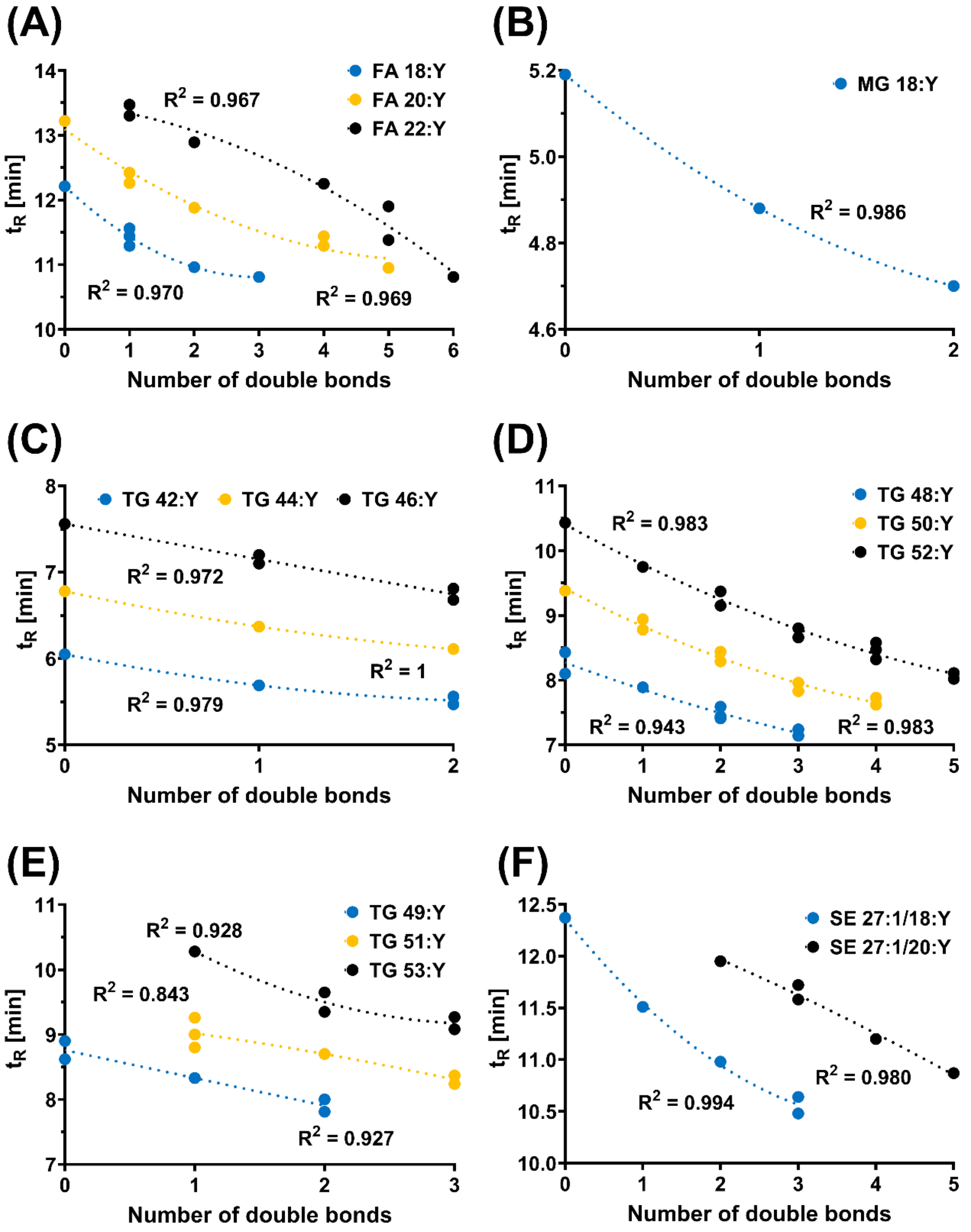

**Figure S10:** Calibration curves of internal standards in spiked human plasma: **(A)** fatty acids, **(B)** monoacylglycerols, **(C)** diacylglycerols, **(D)** triacylglycerols, **(E)** cholesteryl esters, and **(F)** cholesterol. Data represent the mean values from three independent experiments.

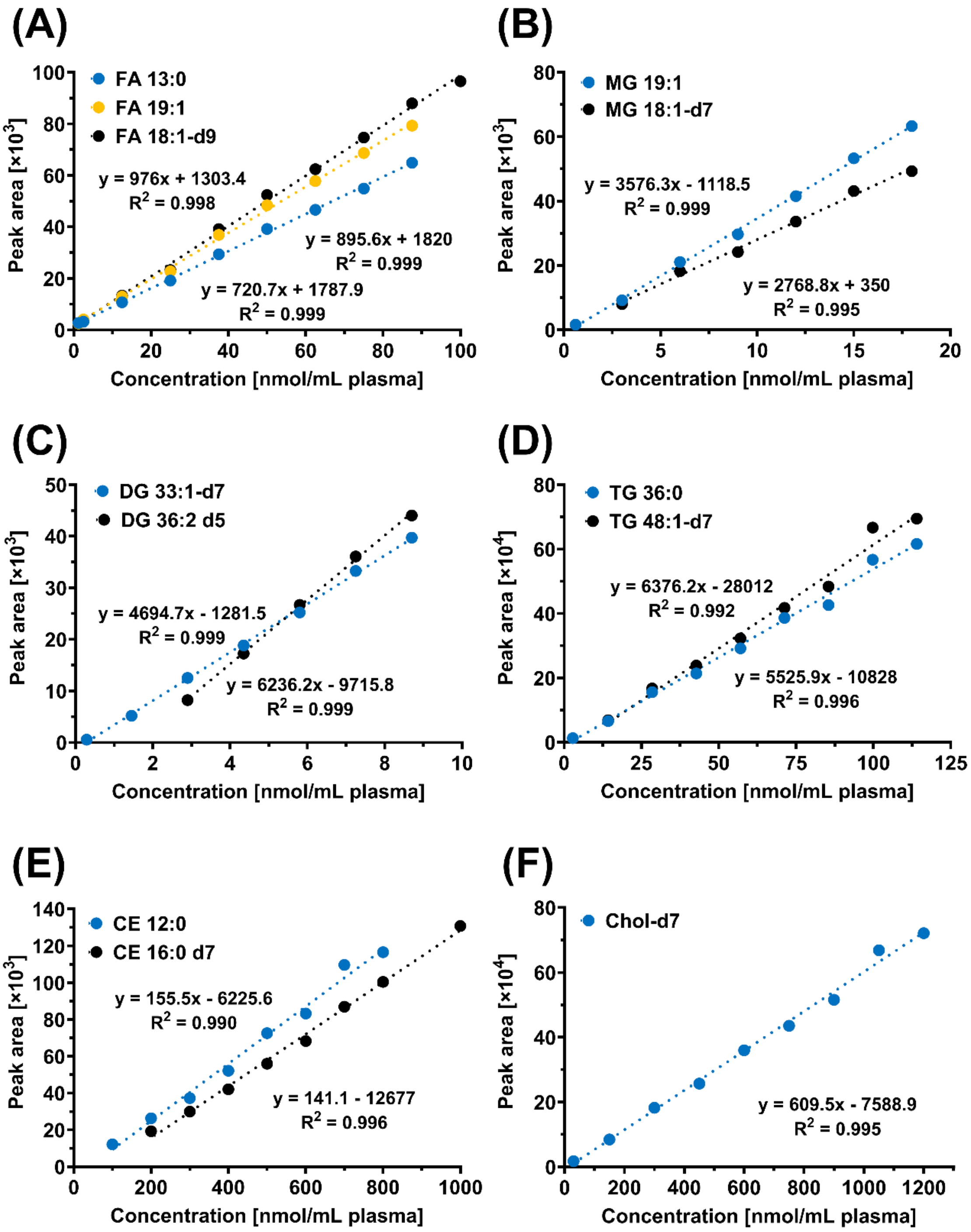

**Figure S11:** Dependences of response factors on fatty acyl chain length for **(A)** saturated and **(B)** monounsaturated cholesteryl esters, and on the number of double bonds for **(C)** C18 and **(D)** C20 fatty acyl chains of cholesteryl esters.

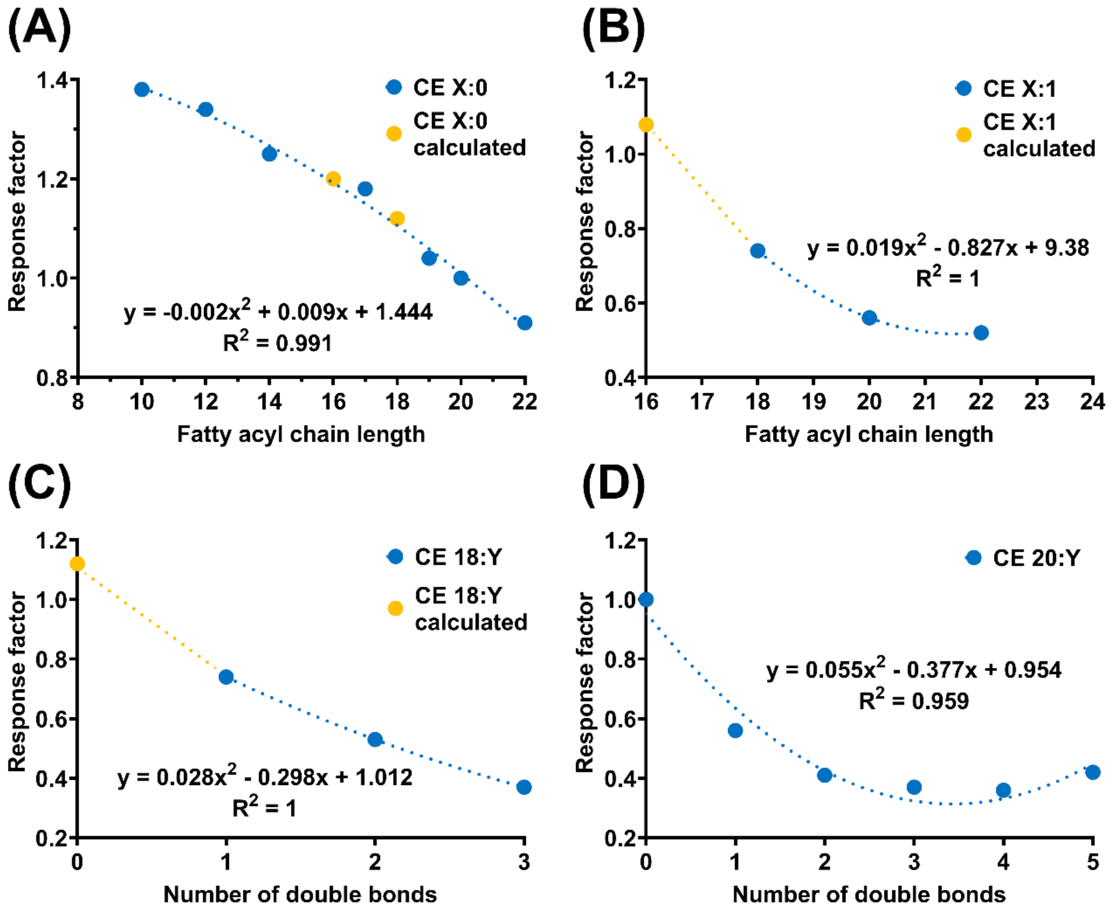

Supplement: Supplementary file 1 [file ac5c04668_si_001.pdf]
